# MYT1L is required for suppressing earlier neuronal development programs in the adult mouse brain

**DOI:** 10.1101/gr.277413.122

**Published:** 2023-04

**Authors:** Jiayang Chen, Nicole A. Fuhler, Kevin K. Noguchi, Joseph D. Dougherty

**Affiliations:** 1 Department of Genetics, Washington University School of Medicine, St. Louis, Missouri 63110, USA;; 2 Department of Psychiatry, Washington University School of Medicine, St. Louis, Missouri 63110, USA;; 3 Intellectual and Developmental Disabilities Research Center, Washington University School of Medicine, St. Louis, Missouri 63108, USA

## Abstract

In vitro studies indicate the neurodevelopmental disorder gene myelin transcription factor 1-like (MYT1L) suppresses non-neuronal lineage genes during fibroblast-to-neuron direct differentiation. However, MYT1L's molecular and cellular functions in the adult mammalian brain have not been fully characterized. Here, we found that MYT1L loss leads to up-regulated deep layer (DL) gene expression, corresponding to an increased ratio of DL/UL neurons in the adult mouse cortex. To define potential mechanisms, we conducted Cleavage Under Targets & Release Using Nuclease (CUT&RUN) to map MYT1L binding targets and epigenetic changes following MYT1L loss in mouse developing cortex and adult prefrontal cortex (PFC). We found MYT1L mainly binds to open chromatin, but with different transcription factor co-occupancies between promoters and enhancers. Likewise, multiomic data set integration revealed that, at promoters, MYT1L loss does not change chromatin accessibility but increases H3K4me3 and H3K27ac, activating both a subset of earlier neuronal development genes as well as *Bcl11b*, a key regulator for DL neuron development. Meanwhile, we discovered that MYT1L normally represses the activity of neurogenic enhancers associated with neuronal migration and neuronal projection development by closing chromatin structures and promoting removal of active histone marks. Further, we showed that MYT1L interacts with HDAC2 and transcriptional repressor SIN3B in vivo, providing potential mechanisms underlying repressive effects on histone acetylation and gene expression. Overall, our findings provide a comprehensive map of MYT1L binding in vivo and mechanistic insights into how MYT1L loss leads to aberrant activation of earlier neuronal development programs in the adult mouse brain.

Neuronal development is a continuous process starting early during embryogenesis and lasting well into the postnatal ages (Stiles and Jernigan 2010; Kroon et al. 2019). Originating from asymmetric divisions of neural progenitors in the ventricular zone (VZ), neurons undergo earlier neuronal development programs, including migration and projection development, followed by later maturation processes, including synaptic development and refinement, to gain their locational and functional identities in different layers of the cortex (Campbell 2005; Götz and Huttner 2005; Luo and O'Leary 2005). These steps are finely tuned by a sophisticated network of *cis*-regulatory elements (e.g., promoters and enhancers), *trans*-regulatory factors (e.g., transcriptional factors [TFs] and histone modifying complexes), as well as epigenetic regulation (e.g., changes in chromatin accessibility and histone modifications) (Olson et al. 2001; Dixit et al. 2014; Lomvardas and Maniatis 2016; Nitarska et al. 2016; Trevino et al. 2020; Yousefi et al. 2021). Thus, proper activation and repression of those early neuronal development programs in a timely manner is crucial for both neuronal generation and later maturation and fate specification (Yuan et al. 2022). TFs, especially proneuronal basic helix-loop-helix (bHLH) TFs, are key components that orchestrate these programs (Olson et al. 2001; Dixit et al. 2014; Tutukova et al. 2021). Several master bHLHs, such as NEUROG2 and NEUROD1, are expressed at different developmental stages and are responsible for facilitating neuronal fate progression by modulating promoters, enhancers, and thus expression of corresponding genes (Dixit et al. 2014; Pataskar et al. 2016; Noack et al. 2022). Although multiomics data set integration has helped identify many of these *cis*- and *trans*-regulatory elements involved in neuronal development, how their activities are precisely controlled to produce mature neuronal cell types remains poorly understood.

In addition to bHLHs, other TFs, including TBR1 and BCL11B, are also developmentally expressed in a cell type–specific manner and play indispensable roles in neuronal development (Arlotta et al. 2008; Bedogni et al. 2010). Myelin transcription factor 1-like (MYT1L), a proneuronal TF expressed primarily in postmitotic neurons, appears to be one such key factor participating in neuronal fate specification and maturation (Mall et al. 2017; Heavner et al. 2020; Chen et al. 2021). For example, in vitro studies by shRNA knockdown suggest that MYT1L loss increases the ratio of deep layer (DL) to upper layer (UL) cortical neurons (Heavner et al. 2020). Furthermore, overexpressing MYT1L, along with ASCL1 and POU3F2 (also known as BRN2), can directly reprogram mouse embryonic fibroblasts (MEFs) into functional neurons, demonstrating its potent roles in promoting neuronal differentiation (Vierbuchen et al. 2010). Utilizing the same reprogramming system, Mall and colleagues mapped MYT1L targets by chromatin immunoprecipitation followed by deep sequencing (ChIP-seq) and measured target gene expression by RNA-seq (Mall et al. 2017). In this system, MYT1L mainly acts as a transcriptional repressor that silences non-neuronal gene expression to facilitate neuronal differentiation (Mall et al. 2017). Meanwhile, other in vitro studies revealed that MYT1L can function as both a transcriptional activator and repressor, probably through distinct protein domains. For example, truncation experiments have shown the N-terminal domain activates gene expression in reporter assays, whereas a central zinc-finger containing domain suppresses expression (Manukyan et al. 2018). However, in the transdifferentiation system, the N-terminus, together with central zinc-finger domains, recruits cofactors including SIN3B and is sufficient for neuronal reprogramming (Mall et al. 2017). SIN3B is thought to then recruit histone deacetylases (HDACs) (Bainor et al. 2018), although direct interactions between MYT1L, SIN3B, and HDACs have not been shown in vivo, nor has MYT1L's impact on histone modifications been examined. Overall, despite widespread usage of MYT1L in neuronal transdifferentiation, the molecular mechanisms underlying MYT1L's proneuronal activities during normal brain development remain poorly defined. Additionally, it is unclear whether MYT1L influences epigenetic landscapes to regulate gene expression.

Recently MYT1L has also been implicated in human neurodevelopmental disorders (NDDs), with the spectrum of symptoms caused by *MYT1L* loss of function (LoF) mutations now recognized as MYT1L Syndrome (Blanchet et al. 2017; Coursimault et al. 2022). To understand MYT1L's functions in vivo and how MYT1L mutations lead to human disease pathology, several in vivo models have been established (Blanchet et al. 2017; Mall et al. 2017; Chen et al. 2021; Wöhr et al. 2022). Knockdown of *MYT1L* homologs (*myt1la* and *myt1lb*) in zebrafish by antisense morpholinos reduces oxytocin and arginine vasopressin mRNA abundance in the hypothalamus, suggesting MYT1L is important for neuroendocrine system development, and/or neuronal maturation, as peptide expression develops relatively late postnatally (Almazan et al. 1989; Blanchet et al. 2017). Furthermore, *Myt1l* shRNA knockdown by in utero electroporation impairs neuronal migration in the developing mouse cortex, echoing its roles in facilitating neuronal development (Mall et al. 2017). As MYT1L Syndrome patients harbor de novo heterozygous mutations of *MYT1L*, more recently a *Myt1l* germline knockout (KO) mouse line was generated to mimic human patient genetics (Chen et al. 2021). This study shows that *Myt1l* heterozygous (Het) KO mice recapitulate many phenotypes reminiscent of the human syndrome, including obesity, hyperactivity, and social deficits. Key phenotypes, like obesity and hyperactivity, are replicated in two additional *Myt1l* haploinsufficiency mouse models (Kim et al. 2022; Wöhr et al. 2022). In the initial *Myt1l* germline KO mouse line, it is also shown that MYT1L loss results in insufficient cell proliferation in embryonic mouse cortex (Chen et al. 2021). Further investigation leveraging existing ChIP-seq data (albeit from in vitro binding experiments [Mall et al. 2017]), along with in vivo ATAC-seq and RNA-seq data sets, reveals MYT1L has a role in activating cell proliferation programs but suppressing early neural differentiation programs. In contrast with predictions of the transdifferentiation system, no obvious activation of non-neuronal lineage genes is found in Het mice (Chen et al. 2021). Yet, one limitation of these analyses is they are based on ChIP-seq data from an orthogonal system. Because ectopic overexpression of MYT1L during transdifferentiation might disrupt normal MYT1L binding activity, high-quality binding profiles of MYT1L in vivo are needed to better understand its functions in physiological conditions. In addition, even though there is sustained expression of MYT1L in the adult brain (Chen et al. 2021), little is known about its functions and binding in later stages of neuronal development, and the long-term consequences of MYT1L loss have not been assessed.

Therefore, we adopted CUT&RUN technology to define MYT1L binding targets in the cortex and investigated its molecular functions through epigenetic profiling and further neuroanatomical studies in vivo. With increased sensitivity from CUT&RUN compared to ChIP-seq (Skene and Henikoff 2017), we aim to provide a comprehensive map of MYT1L binding in the mammalian brain and define long-term consequences of MYT1L loss on molecular and cellular levels.

## Results

### MYT1L loss alters the ratio of deep/upper layer neurons in mouse cortex

**Figure 1.**
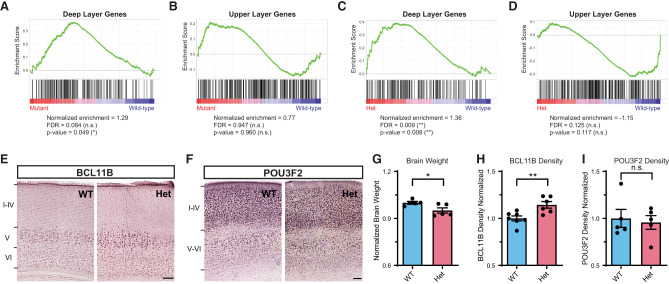
MYT1L controls cortical neuron layer specification. (*A*) GSEA showed an up-regulation of DL genes in *Myt1l* mutant E14 CTX. (*B*) UL genes showed no significant change in *Myt1l* mutant E14 CTX. (*C*) GSEA showed an up-regulation of DL genes in *Myt1l* Het P60 PFC. (*D*) UL genes showed subtle but not significant down-regulation in *Myt1l* Het P60 PFC. (*E*) Representative images of DL neuronal marker BCL11B staining on the P60 mouse cortex. (*F*) Representative images of UL neuronal marker POU3F2 staining on the P60 mouse cortex. (*G*) *Myt1l* Het mice had reduced brain weights compared to WTs. (*H*) *Myt1l* Het mice had increased BCL11B^+^ neuron density in cortex. (*I*) POU3F2^+^ neuron density remains the same between Hets and WTs. (*) *P* < 0.05, (**) *P* < 0.01. Data were represented as Mean ± SEM. Scale bar, 100 µM.

A previous in vitro study has shown that *Myt1l* knockdown increased the ratio of DL/UL neurons (Heavner et al. 2020). Thus, we first investigated whether *Myt1l* constitutive heterozygous (Het) KOs, which mimic MYT1L Syndrome patients’ gene dose, can also result in similar phenotypes in mice. First, we re-evaluated the RNA-seq data set from *Myt1l* mutant (Het and homozygous KO) embryonic day 14 cortex (E14 CTX) and *Myt1l* Het PFC (Chen et al. 2021). With gene set enrichment analysis (GSEA), we found there is a significantly increased expression of DL neuron signature genes in mutant E14 CTX compared with wild-type (WT) (Fig. 1A). Expression of the UL neuron signature genes is unchanged (Fig. 1B); however, as UL genes are not yet highly expressed by E14, this finding may not be conclusive. Therefore, we also examined adult PFC when both neuronal subtypes are present. In the adult Het PFC, the expression of DL genes is even more significantly up-regulated (Fig. 1C), and UL genes again show no significant down-regulation (Fig. 1D), suggesting that the impact of MYT1L loss on DL gene expression is not a transient effect. As DL neurons are in an earlier neuronal development trajectory than UL neurons, up-regulation of DL neuron genes on MYT1L loss is consistent with the hypothesis that normal MYT1L levels are needed to facilitate neuronal maturation.

One possible explanation for the adult RNA-seq pattern is that Hets have more DL neurons than WT controls. Thus, to test this hypothesis and examine MYT1L's role in regulating neuronal localization in vivo, we stained the postnatal day 60 (P60) Het cortex (Fig. 1E,F) with UL and DL markers. As expected, we replicated the finding that Het mice have reduced brain weights compared with WT littermates (Fig. 1G; Chen et al. 2021). Consistent with our hypothesis, we found Het cortices have increased DL neuron density (labeled by BCL11B [also known as CTIP2] counted from cortical layer V-VI) compared with WT littermates (Fig. 1E,H). On the other hand, UL neurons (labeled by POU3F2 [also known as BRN2] counted from cortical layer I-IV) did not show altered density in Het cortices (Fig. 1F,I). Together, both RNA-seq and immunochemistry validation experiments showed MYT1L loss altered the ratio of DL/UL neurons in the mouse cortex.

### CUT&RUN identifies MYT1L binding targets in the mouse embryonic cortex

**Figure 2.**
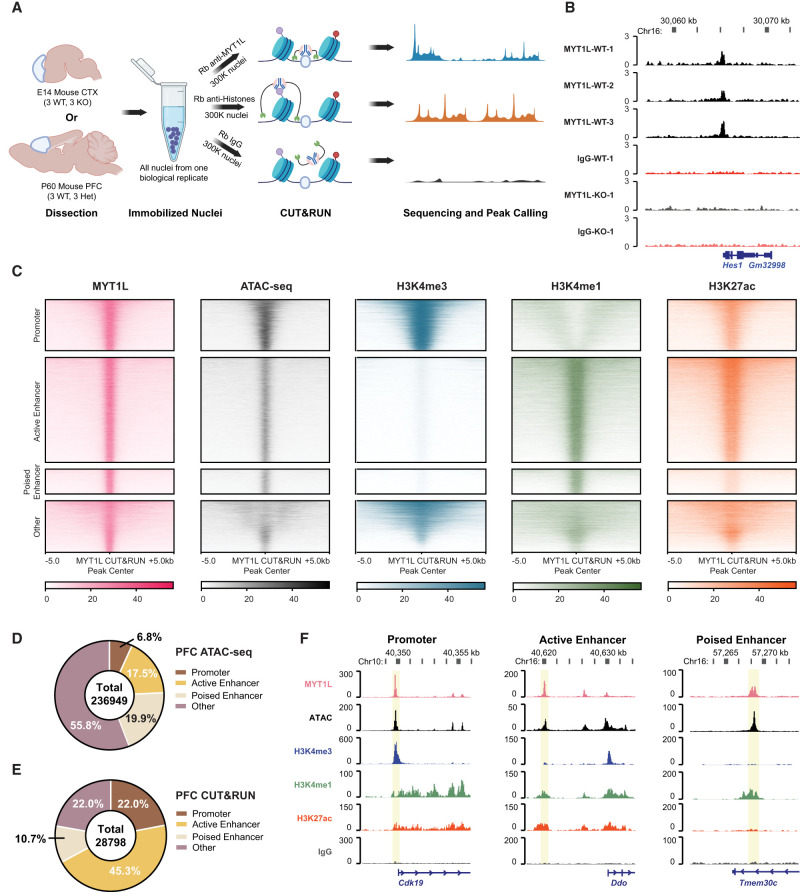
CUT&RUN identifies MYT1L-specific binding targets in E14 mouse cortex and adult mouse PFC. (*A*) Workflow of CUT&RUN experiments on E14 CTX and adult prefrontal cortex (PFC) created with BioRender (https://www.biorender.com/). (*B*) Representative Integrative Genomics Viewer (IGV) tracks showing a reproducible MYT1L peak at the *Hes1* promoter region in all 3 WT E14 biological replicates but not in IgG and KO samples. (*C*) Heatmaps of CUT&RUN signals of MYT1L, IgG, and histones at MYT1L-bound regions in PFC. (*D*) Annotations of PFC ATAC-seq peaks showed the genome-wide distribution of promoters, active enhancers, and poised enhancers in open chromatin regions. (*E*) Annotations of MYT1L targets in PFC showed MYT1L mainly binds to active enhancers. (*F*) Representative genome tracks of MYT1L-bound promoter (*left*), active enhancer (*middle*), and poised enhancer (*right*).

To understand how MYT1L regulates the DL/UL neuron ratio, as well as the altered transcriptional profile we previously observed (Chen et al. 2021), we next investigated MYT1L genomic binding targets in vivo. MYT1L has peak protein expression between E14 and P1 in the mouse brain (Chen et al. 2021). In order to map MYT1L targets in vivo, we optimized CUT&RUN on E14 mouse cortex because we had the benchmark multiomics data for potential integration from previous studies (Fig. 2A; Skene and Henikoff 2017; Chen et al. 2021). First, leveraging the *Myt1l* germline KO (S710fsX) mouse line (Chen et al. 2021), we validated CUT&RUN and antibody specificity on *Myt1l* KO samples. These S710fsX KO mice do not produce any MYT1L protein and thus can serve as a gold-standard control (Chen et al. 2021). Indeed, we identified 560 MYT1L peaks in WT E14 mouse cortex, whereas no peak was called in KO samples (Fig. 2B; Supplemental Table S1), indicating excellent antibody specificity. In addition, in de novo motif finding, the known MYT1L core binding motif AAGTT (Jiang et al. 1996; Mall et al. 2017) was significantly enriched in all 560 peaks (100% of targets, *P* = 1 × 10^−11^) (Supplemental Fig. S1A). These results suggest CUT&RUN can be applied to profile MYT1L binding activities in the mouse cortex with great specificity. Because MYT1L binding profiles have not been well characterized in postnatal brains, we next conducted CUT&RUN on the adult mouse brain. The E14 brain has relatively few MYT1L-expressing cells (postmitotic neurons) (Chen et al. 2021), thus the adult brain with its higher neuron proportion may map MYT1L binding in a more efficient way.

### Using adult mouse prefrontal cortex improves CUT&RUN sensitivity on MYT1L profiling

To better compare with existing multiomics data sets and human phenotypes, we chose adult mouse PFC as the target region for MYT1L CUT&RUN. This brain region is associated with attention deficit and hyperactivity disorder (ADHD) (Yasumura et al. 2019), which is observed in MYT1L Syndrome human patients, and hyperactivity is found in the mouse models. First, we identified 28,798 reproducible MYT1L-bound peaks across three biological replicates of WT mouse PFC (Fig. 2C; Supplemental Table S1). The MYT1L core binding motif AAGTT is significantly enriched via de novo motif finding (76.37% of targets, *P* = 1 × 10^−3125^) (Supplemental Fig. S1B). Overall, many more peaks were identified from the adult PFC CUT&RUN experiments compared with E14 cortex, and the majority of the E14 peaks were also recovered in PFC CUT&RUN (Supplemental Fig. S1C,D), suggesting CUT&RUN on adult PFC might be more efficient than E14 or MYT1L might have more binding activities in adults. In addition, we compared our MYT1L CUT&RUN targets with MYT1L ChIP-seq data from both E14 brain and MEFs overexpressing MYT1L, POU3F2, and ASCL1 (Mall et al. 2017). We did not see significant overlap between MYT1L CUT&RUN and either of the ChIP-seq data sets (Supplemental Fig. S2A–H). However, for the targets that overlap between PFC CUT&RUN and E14 mouse brain ChIP-seq, they tend to have higher peak enrichment scores compared to non-overlapped targets (Supplemental Fig. S2I,J), and their peak enrichment scores also have subtle but significant positive correlation (Supplemental Fig. S2K). Therefore, both CUT&RUN and ChIP-seq can identify some common strong MYT1L binding activities in the genome. However, targets that overlap between PFC CUT&RUN and MEF ChIP-seq only showed significantly higher peak enrichment in the CUT&RUN data but not in ChIP-seq (Supplemental Fig. S2L,M), and they failed to show any correlation between two techniques (Supplemental Fig. S2N). Considering the mouse PFC is more related to E14 mouse brains than to MEFs, these results suggest MYT1L binding might be context dependent with different binding in different cell types and developmental time points. Thus, MYT1L likely does not serve as a pioneer factor that opens the chromatin of its targets in any cellular context. Meanwhile, both MYT1L CUT&RUN and ChIP-seq on the embryonic mouse brain identified a limited number of MYT1L targets, making it challenging to perform a well-powered comparison. A more sensitive and efficient profiling method is needed for detecting MYT1L binding during early brain development. Because data from PFC have more peaks and better MYT1L motif enrichment significance compared to E14 CUT&RUN, we focused on the 28,798 MYT1L binding targets as well as epigenetic profiles identified from adult PFC CUT&RUN for the downstream analysis to understand MYT1L's functions in the adult brain and the long-term consequences of MYT1L loss.

### MYT1L co-occupies its binding sites with different transcription factors at promoter and enhancer regions

**Figure 3.**
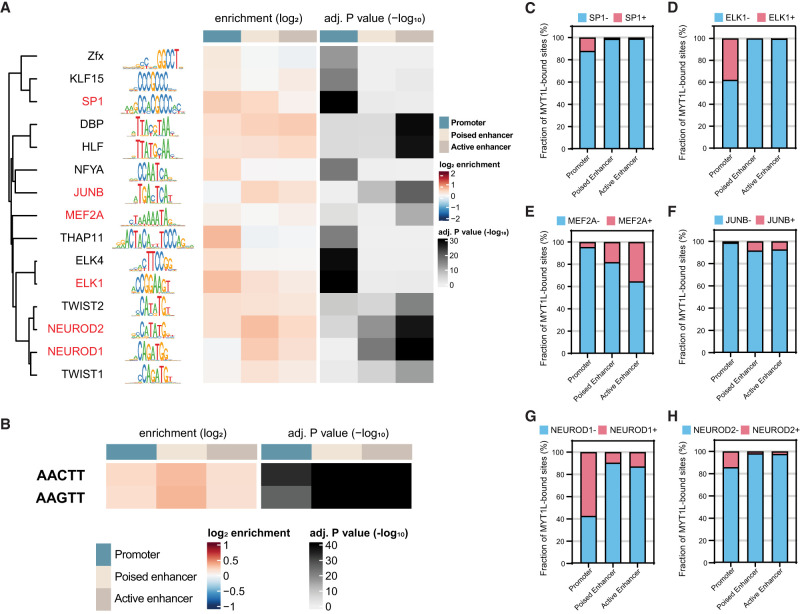
MYT1L co-occupies with different sets of TFs at promoter and enhancer regions. (*A*) monaLisa motif analysis revealed that MYT1L co-occupies with transcriptional activators such as ELK1 at promoter regions, whereas it co-occupies with neurogenic TFs such as MEF2A at enhancer regions. (*B*) Both MYT1L-bound promoters and enhancers were significantly enriched for MYT1L core binding motif, AAGTT. (*C*) Overlapping between MYT1L CUT&RUN targets and TFs ChIP-seq peaks showed that more MYT1L promoter targets were also bound by transcriptional activators like SP1 and (*D*) ELK1 than enhancer targets, whereas more enhancer targets were bound by (*E*) the neurogenic TF MEF2A and (*F*) activity-dependent protein JUNB. (*G*) NEUROD1 and (*H*) NEUROD2 had stronger presence at MYT1L promoter targets than enhancer targets.

Previous ChIP-seq experiments have shown that MYT1L mainly binds to the promoter regions when overexpressed during reprogramming of MEFs (Mall et al. 2017). To test if this is also true in vivo, we annotated MYT1L targets from adult mouse PFC. Analysis of MYT1L colocalization at these regions showed that MYT1L tends to bind open chromatin regions (95.3%, 27,450/28,798) with enhancers being the most common category when compared to all open chromatin regions (Fig. 2D–F; Supplemental Table S2). Meanwhile, we used nuclei from the same animals to perform CUT&RUN on several histone modifications, including H3K4me3, H3K4me1, and H3K27ac (Fig. 2A). Leveraging these histone modification profiles, we further categorized enhancers (Supplemental Table S3) into poised (H3K4me1+/H327ac−) and active (H3K4me1+/H3K27ac+) enhancers as previously described (Fig. 2C,F; Creyghton et al. 2010). To understand sequence preferences of MYT1L at promoters, poised enhancers, and active enhancers, we performed motif analysis using monaLisa to compare de novo binding motifs and predicted TF co-occupancies between the three (Machlab et al. 2022). To control baseline abundance of certain TF motifs at promoters or enhancers, we used all promoters, poised enhancers, and active enhancers detected in the PFC as the motif analysis background for the corresponding genomic regions bound by MYT1L (e.g., MYT1L^+^ promoters over all promoters). We found motifs for TFs that behave as transcriptional activators (e.g., SP1 and ELK1) to be enriched in MYT1L-bound promoters, whereas neurogenic TF (e.g., MEF2A and NEUROD1) and activity-dependent TF (e.g., JUNB) motifs were specifically enriched in MYT1L-bound enhancers (Fig. 3A), even when controlling for the differential baseline frequency of these TF motifs at promoters, poised enhancers, and active enhancers, respectively. Furthermore, differential motif usage is not driven by the MYT1L core binding motif AAGTT because both MYT1L-bound promoters and enhancers are significantly enriched for AAGTT (Fig. 3B).

In order to investigate if these motif abundances reflect actual TF co-occupancy, we compared MYT1L CUT&RUN targets with published TF ChIP-seq data. As expected, compared to MYT1L-unbound regions (MYT1L^−^), ChIP-seq peaks of candidate TFs, including SP1, ELK1, MEF2A, JUNB, NEUROD1, and NEUROD2, are significantly enriched in MYT1L-bound (MYT1L^+^) genomic regions (Supplemental Fig. S3A–G), suggesting these TFs are frequently found at MYT1L-bound peaks. Next, we compared TF ChIP-seq enrichment between the MYT1L^+^ promoters and the MYT1L^+^ enhancers because the motif presence does not necessarily mean actual TF binding. Echoing the motif analysis, MYT1L has significantly higher co-occupancy with SP1 and ELK1 in promoter regions than in enhancer regions (Fig. 3C,D; Supplemental Fig. S3G). Likewise, MEF2A and JUNB prefer binding MYT1L enhancer targets over promoter targets (Fig. 3E,F; Supplemental Fig. S3G). However, in contrast to the motif analysis, a much higher percentage of bHLH TFs NEUROD1 and NEUROD2 ChIP-seq peaks are at MYT1L-bound promoter targets than at MYT1L-bound enhancers (Fig. 3G,H; Supplemental Fig. S3G), suggesting relative motif enrichment will not always predict the corresponding TF binding, or that a different protein which shares similar binding motif might be binding this sequence at enhancers. Meanwhile, MYT1L does not appear to block the binding of NEUROD1 and NEUROD2 at enhancers because no obvious depletion of TF binding in MYT1L^+^ enhancers was observed (Supplemental Fig. S3E,F). Overall, such differential TF co-occupancy suggests MYT1L might play different roles at promoters and enhancers by cooperating with different cofactors.

### MYT1L directly binds to promoters of genes involved in earlier neuronal development

**Figure 4.**
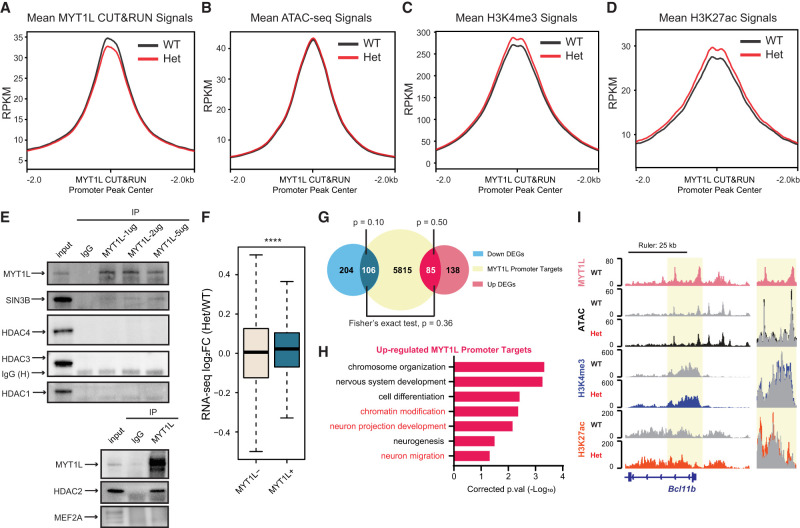
MYT1L directly binds to and controls promoters that are associated with early neuronal development genes. (*A*) Mean MYT1L CUT&RUN signals showed decreased MYT1L binding at promoters in Het PFC. (*B*) No chromatin accessibility change was observed at MYT1L-bound promoters. (*C*) Mean H3K4me3 CUT&RUN signals showed increased H3K4me3 at MYT1L-bound (MYT1L^+^) promoters in Het PFC. (*D*) Mean H3K27ac CUT&RUN signals showed increased H3K27ac at MYT1L-bound promoters in Het PFC. (*E*) MYT1L coimmunoprecipitated with SIN3B and HDAC2 but not with HDAC1/3/4 and MEF2A in WT mouse cortex. (*F*) MYT1L loss increased its promoter targets’ expression in PFC. (****) *P* < 0.0001. (*G*) Venn diagram showing the overlaps among dDEGs, MYT1L promoter targets, and uDEGs. No biased overlap was observed (*P* = 0.36). (*H*) GO analysis on 85 uDEGs whose promoters were bound by MYT1L. (*I*) Representative genome browser track showed MYT1L-bound *Bcl11b* promoter had higher H3K4me3 and H3K27ac levels in Het PFC than WT.

To understand molecular functions of MYT1L binding, we first focused on MYT1L-bound promoters because they can be directly associated with nearby genes, thus allowing functional inferences of downstream consequences. Chromatin accessibility is closely correlated with gene expression, in which open chromatin indicates active gene expression, whereas closed chromatin indicates gene repression. Therefore, we compared MYT1L binding data with Assay of Transposase Accessible Chromatin sequencing (ATAC-seq) on P60 mouse PFC (Het vs. WT) (Chen et al. 2021) to see whether MYT1L binding changes chromatin structure at promoters. We found that MYT1L haploinsufficiency indeed reduces overall MYT1L binding activity, but does not affect chromatin accessibility at promoter targets (Fig. 4A,B; Supplemental Fig. S4A,B). Histone modifications are also closely correlated with gene expression, with H3K4me3 and H3K27ac thought to be marks of active gene expression (Heintzman et al. 2009; Black et al. 2012). Thus, we investigated how the histone landscape changes at promoter regions on MYT1L loss. We found there were more H3K4me3 and H3K27ac modifications in Het PFC at MYT1L promoter targets compared with WT (Fig. 4C,D; Supplemental Fig. S4C,D), indicating MYT1L's role is normally to suppress gene expression by facilitating the removal of these marks. Previous in vitro studies have shown MYT1L can bind to SIN3B, a transcriptional repressor that recruits histone deacetylase (HDAC1/2) and demethylase (KDM5A) (Naruse et al. 1999; Romm et al. 2005; Hayakawa et al. 2007; Nishibuchi et al. 2014; Bainor et al. 2018). To explore potential mechanisms underlying MYT1L modifying chromatin landscapes, we screened several MYT1L cofactor candidates using coimmunoprecipitation (Co-IP) according to previous studies (Romm et al. 2005; Mall et al. 2017) and motif analysis. We found MYT1L interacts with SIN3B as well as HDAC2 in the mouse cortex, whereas no direct interaction between MYT1L and HDAC1, HDAC3, HDAC4, or MEF2A was observed (Fig. 4E). These results suggest that although MYT1L has minimal effects on chromatin accessibility at its bound promoters, it can facilitate the removal of active histone marks at promoters, potentially via interacting with the SIN3B repressor complex.

We next sought to determine the impact of these epigenetic changes at promoters on gene expression. By looking at adult Het PFC RNA-seq fold changes of genes whose promoters are bound by MYT1L, we saw a subtle but significant up-regulated expression of those MYT1L promoter targets in Het (Fig. 4F), echoing MYT1L's role as a transcriptional repressor in reprogramming neurons (Mall et al. 2017). Then, we specifically looked at differentially expressed genes (DEGs). Among 309 down-regulated genes (dDEGs) and 223 up-regulated genes (uDEGs) in MYT1L Het PFC, we saw an unbiased distribution of MYT1L promoter targets, with 106 dDEGs' and 85 uDEGs’ promoters bound by MYT1L (Fig. 4G). Meanwhile, there was no significant overlap between MYT1L promoter targets and dDEGs or uDEGs, indicating the majority of transcriptomic changes were caused by indirect effects of MYT1L loss (Fig. 4G). Gene Ontology (GO) analysis on these overlapped genes revealed that MYT1L promoter targets up-regulated in Hets are significantly enriched in chromatin modification (e.g., *Hdac4*, Supplemental Fig. S4E) and neuron projection development (e.g., *Lingo1* and *Cit*) pathways (Fig. 4H). Notably, promoters of several key regulators of neuronal migration (e.g., *Ctnnd2*, Supplemental Fig. S4F) and deep cortical layer identity genes are directly bound by MYT1L, and the corresponding genes were up-regulated in Het PFC, including *Bcl11b*, a master regulator of DL neuronal fate (Fig. 4I; Arlotta et al. 2005). However, mature neuron functional pathways, like synaptic transmission and ion transport, which were down-regulated in Het PFC (Chen et al. 2021), are not enriched in MYT1L promoter targets (Supplemental Table S4), indicating their dysregulation is likely an indirect effect of MYT1L loss. Together, these findings suggest that MYT1L directly suppresses earlier neuronal development programs like neuronal migration and projection by altering corresponding promoters’ epigenetics in the adult mouse PFC. In addition, MYT1L loss increases H3K4me3 and H3K27ac at the promoter of DL marker gene, *Bcl11b*, and up-regulates its expression, providing a molecular mechanism underlying the increased DL neuron numbers.

### MYT1L represses enhancers that control earlier neuronal development programs

**Figure 5.**
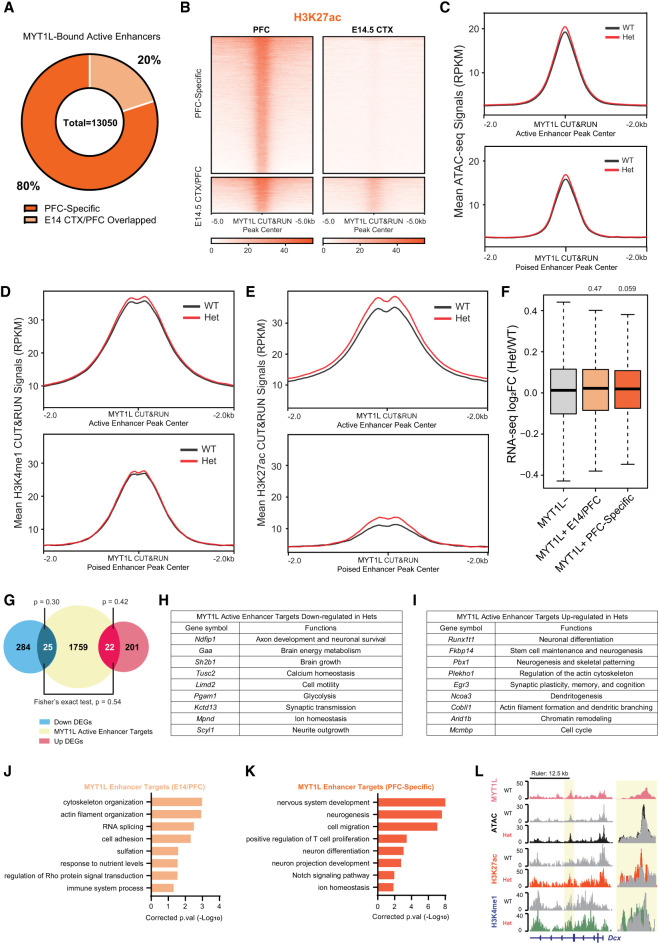
MYT1L suppresses enhancers that regulate neuronal migration and neuron projection development. (*A*) Majority of MYT1L-bound active enhancers were PFC-specific. (*B*) Heatmaps of MYT1L-bound active enhancers in PFC. (*C*) MYT1L loss increased its bound active enhancers but not poised enhancers’ chromatin accessibility. (*D*) MYT1L loss increased H3K4me1 levels at MYT1L-bound active enhancers but did not significantly increase H3K4me1 levels at MYT1L-bound poised enhancers. (*E*) MYT1L loss increased both its bound active and poised enhancers’ H3K27ac levels. (*F*) MYT1L active enhancer target genes showed increased expression in Het PFC. (*G*) Venn diagram showing the overlaps among dDEGs, MYT1L active enhancer targets, and uDEGs. There were more overlaps between MYT1L active enhancer targets and uDEGs than dDEGs (*P* = 0.01). (*H*) Functions of MYT1L active enhancer targets showing down-regulation in RNA-seq. (*I*) Functions of MYT1L active enhancer targets showing up-regulation in RNA-seq. (*J*) GO analysis on genes associated with MYT1L-bound (MYT1L^+^) E14 CTX/PFC overlapped active enhancers. (*K*) GO analysis on genes associated with MYT1L^+^ PFC-specific active enhancers. (*L*) Representative genome browser track showed MYT1L-bound *Dcx* active enhancer has higher ATAC-seq signals, H3K4me1 and H3K27ac levels in Het PFC than WT.

Previous studies have shown enhancers are crucial for controlling neurodevelopmental programs as well as neuronal functions (Malik et al. 2014; Lu et al. 2020). Given enhancers have spatiotemporal activities throughout development (Carullo and Day 2019), and most MYT1L targets identified in PFC are enhancers, we investigated how MYT1L binding influences enhancer epigenetics. After categorizing enhancers into active and poised stages by H3K27ac enrichment (Fig. 3C), we found MYT1L preferentially binds to activated enhancers compared to poised enhancers in the adult mouse PFC (Fig. 3E), and that the Het PFC has reduced MYT1L binding at both active and poised enhancers (Supplemental Fig. S5A). Next, we explored how MYT1L-bound enhancers are developmentally regulated. By integrating the histone CUT&RUN data from E14 CTX, we defined all active and poised enhancers, regardless of MYT1L binding, in E14 CTX and compared those with all adult PFC enhancers. Out of 13,050 active enhancers bound by MYT1L in adult PFC, 80% (10,443/13,050) are adult-specific active enhancers (PFC-specific), and only 20% are characterized as active enhancers at both developmental time points (E14 CTX/PFC Overlapped, Fig. 5A,B). Furthermore, 24.8% (2593/10,443) of those adult-specific active enhancers bound by MYT1L were poised in E14 CTX (Supplemental Table S2), suggesting MYT1L might also guide the activation of a small subset of poised enhancers during development. On the other hand, out of 3077 MYT1L targets annotated as E14 CTX active enhancers, only 21.3% (656/3077) of them were E14 CTX-specific active enhancers (Supplemental Table S2). This suggests that MYT1L occupancy at active enhancers is more prevalent in the adult stage, consistent with its major expression pattern in postmitotic neurons (Chen et al. 2021).

Next, we examined chromatin structure and histone landscape alterations at MYT1L-bound enhancers in Het PFC to understand how MYT1L regulates enhancer activity. Unlike MYT1L-bound promoters which showed no ATAC differences, MYT1L-bound active enhancers showed increased chromatin accessibility in Hets, compared to WTs (Fig. 5C; Supplemental Fig. S5B), whereas MYT1L-bound poised enhancers have no change (Fig. 5C; Supplemental Fig. S5B). When looking only at differential accessible regions (DARs, defined in Chen et al. [2021]) annotated as active enhancers, up-regulated DARs (DARs with increased chromatin accessibility in Hets) have a higher percentage in MYT1L-bound active enhancers than in MYT1L-active enhancers (Supplemental Fig. S5C). DARs annotated as poised enhancers showed the same pattern (Supplemental Fig. S5D). This shows that, at least on the chromatin accessibility level, MYT1L tends to close the chromatin of its bound enhancers, and many DARs are direct effects. To better understand the enhancer activities, we looked into active histone marks, including H3K4me1 and H3K27ac. MYT1L-bound active enhancers have increased H3K4me1 levels in Hets compared to WTs, whereas MYT1L-bound poised enhancers showed unchanged H3K4me1 (Fig. 5D; Supplemental Fig. S5E). Notably, both MYT1L-bound active and poised enhancers displayed increased enrichment of H3K27ac, a histone modification marking enhancer activation in Het PFC (Fig. 5E; Supplemental Fig. S5F), suggesting MYT1L loss can activate both its bound poised and active enhancers. Together, these results indicate that MYT1L normally facilitates repression of its bound enhancers, and MYT1L loss leads to aberrant enhancer opening and activation.

Enhancers are important *cis*-regulatory elements for gene expression. Therefore, we again leveraged our RNA-seq data sets to understand how MYT1L together with enhancers control gene expression and to define the transcriptional consequences of MYT1L loss at enhancers. To find enhancer–gene pairs (“enhancer targets,” Supplemental Table S5), we used EnhancerAtlas 2.0, a consensus enhancer prediction database based on multiple high-throughput data sets, including histone modifications, ATAC-seq, ChIA-seq, etc. (Gao and Qian 2020). We focused only on active enhancers because they are MYT1L's major targets, and we identified a total of 1806 active enhancer targets. Although MYT1L E14 CTX/PFC overlapped enhancer targets showed no significant expression change, MYT1L PFC-specific active enhancer targets had a subtly increased expression compared to all other genes that are not regulated by MYT1L, consistent with their increased active histone marks (Fig. 5F). When overlapping with DEGs, there are few DEGs associated with MYT1L-bound active enhancers (Fig. 5G). We further looked into functions of those DEGs whose enhancers are bound by MYT1L. We found down-regulated MYT1L active enhancer targets in Hets tend to be associated with synaptic transmission (e.g., *Ndfip1*, *Kctd13*, and *Mpnd*), whereas up-regulated MYT1L active enhancer targets are likely associated with earlier neuronal development (e.g., *Runx1t1*, *Pbx1*, and *Cobll1*, Fig. 5H,I). These results indicate that the neuronal immaturity seen in the adult Het PFC (Chen et al. 2021) might also be directly associated with disrupted activities of MYT1L-bound active enhancers.

However, with a limited number of overlaps between MYT1L active enhancer targets and DEGs, we could not use GO analysis to have a broader view of MYT1L-bound enhancers’ functions. Therefore, we performed GO analysis on all MYT1L active enhancer targets regardless of corresponding gene expression. GO analysis on MYT1L E14 CTX/PFC overlapped active enhancer targets displayed enrichment of cytoskeleton pathways and responses to nutrients (Fig. 5J). This is consistent with cytoskeleton and nutrient intake related biological processes being constantly required from early development to adulthood. Meanwhile, PFC-specific active enhancer targets showed significant enrichment of neuronal migration and projection pathways (e.g., *Dcx*, Fig. 5K,L), indicating the activation of earlier neuronal development programs in Het PFC seen at promoters can also be further exacerbated by dysregulation of MYT1L-bound active enhancers.

### MYT1L loss alters histone modification landscapes at specific target loci

The above sections revealed that MYT1L might play an important role in shaping histone modification landscapes in the adult mouse PFC. To further pinpoint specific loci influenced by MYT1L binding and histone changes, we performed differential enrichment analysis on H3K4me3, H3K4me1, and H3K27ac, and identified genomic regions displaying differential enrichment for these histone marks. We first looked at H3K4me3, the active histone mark for promoters. We identified 1110 differentially enriched H3K4me3 peaks (diff-H3K4me3 peaks) in Het PFC, and 41.0% of them (455/1110) are bound by MYT1L (MYT1L^+^) (Supplemental Fig. S6A,B). Notably, compared to non-MYT1L (MYT1L^−^) diff-H3K4me3 peaks, MYT1L-bound diff-H3K4me3 peaks have a higher percentage of up-regulated peaks than down-regulated peaks (Supplemental Fig. S6B), suggesting a primary repressive role of MYT1L on H3K4me3. GO analysis on genes associated with MYT1L-bound diff-H3K4me3 peaks revealed a potential down-regulation of neurotransmitter secretion and cell cycle pathways and an up-regulation of earlier neuronal development pathways (Supplemental Fig. S6C,D), echoing published RNA-seq findings (Chen et al. 2021). In addition, no ATAC-seq signal change was found in MYT1L-bound diff-H3K4me3 regions in Het PFC, again indicating MYT1L loss likely does not drastically affect chromatin accessibility at its bound promoters (Supplemental Fig. S6E,F). After mapping to DEGs, we found the cell proliferation gene, *Fbxo2*, shows down-regulation in both H3K4me3 enrichment and RNA-seq, whereas earlier neuronal development genes (e.g., *Dlx2*) show up-regulation in both data sets (Supplemental Fig. S6G,H). These results suggest MYT1L mainly functions as a repressor on H3K4me3 to regulate earlier neuronal development programs.

**Figure 6.**
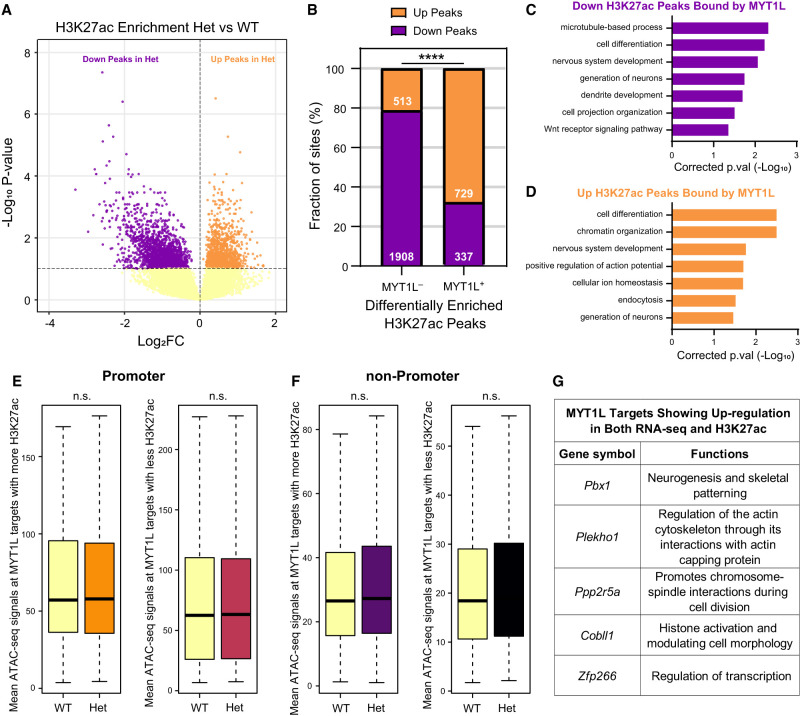
MYT1L loss alters the H3K27ac landscape across the genome. (*A*) Volcano plot showing differential enrichment analysis identified 3487 diff-H3K27ac peaks in Het PFC. (*B*) Distribution of down- and up-regulated diff-H3K27ac peaks within the non-MYT1L (MYT1L^−^) and MYT1L-bound (MYT1L^+^) categories. (****) *P* < 0.0001. (*C*) GO analysis on down-regulated diff-H3K27ac peaks bound by MYT1L and (*D*) up-regulated diff-H3K27ac peaks bound by MYT1L. (*E*) Mean ATAC-seq signals of MYT1L^+^ diff-H3K27ac promoter peaks (*left*: up-regulated peaks, *right*: down-regulated peaks). (*F*) Mean ATAC-seq signals of MYT1L^+^ diff-H3K27ac non-promoter peaks (*left*: up-regulated peaks, *right*: down-regulated peaks). (*G*) Functions of MYT1L targets showing up-regulation in both RNA-seq and H3K27ac.

Next, we performed the same analysis on H3K4me1, the histone modification that marks enhancers. Similarly, among 5611 differentially enriched H3K4me1 (diff-H3K4me1) peaks in Het PFC (Supplemental Fig. S7A), MYT1L-bound diff-H3K4me1 peaks have a higher percentage of up-regulated peaks in Hets compared to MYT1L^−^ diff-H3K4me1 peaks (Supplemental Fig. S7B). However, GO analysis only resulted in generic biological process pathways associated with MYT1L-bound diff-H3K4me1 peaks (Supplemental Fig. S7C,D), and ATAC-seq signals remain unchanged in these regions (Supplemental Fig. S7E,F). When integrating with DEGs, there are several cytoskeleton genes (e.g., *Plekho1*) that showed significant changes in both H3K4me1 enrichment and gene expression (Supplemental Fig. S7G,H). This suggests that MYT1L also tends to primarily act like a repressor on H3K4me1, but the downstream consequences are inconclusive. Therefore, we next sought to investigate enhancer activities in a more sensitive way by assessing differential enrichment of H3K27ac, a histone modification that marks active enhancers. We defined 3487 differentially enriched H3K27ac (diff-H3K27ac) peaks with 30.6% (1066/3487) bound by MYT1L (Fig. 6A,B). Again, the majority of MYT1L-bound diff-H3K27ac peaks are up-regulated in Het PFC (Fig. 6B), emphasizing MYT1L's repressive effects on H3K27ac reported in previous metagene analysis. Meanwhile, both down- and up-regulated MYT1L-bound diff-H3K27ac peaks are associated with neuronal differentiation and ion homeostasis pathways (Fig. 6C,D), and there were no ATAC-seq signal differences between WT and Het (Fig. 6E,F). Although no MYT1L-bound down-regulated DEGs were found to have significantly less H3K27ac enrichment, we identified multiple MYT1L-bound up-regulated DEGs displaying more enriched H3K27ac in Het PFC. These genes are associated with cytoskeleton patterning (e.g., *Plekho1*) and earlier neuronal development programs (e.g., *Pbx1* and *Cobll1*) (Fig. 6G). It was unsurprising to see an up-regulation of cytoskeletal programs along with elevated neuronal projection programs because the cytoskeleton is indispensable for neuronal projection development (Kawauchi and Hoshino 2008; Heng et al. 2010). These results again suggest MYT1L normally can suppress earlier neuronal development programs via decreasing H3K27ac. By leveraging differential enrichment analysis on histone modifications, we further defined that MYT1L more often functions as a repressor on H3K4me3, H3K4me1, and H3K27ac. Furthermore, we showed histone landscape changes might be directly implicated in the aberrant activation of earlier neuronal programs upon MYT1L loss in the adult mouse brain.

## Discussion

Here, we used a *Myt1l* germline KO mouse model to investigate MYT1L's role in neuronal maturation and the underlying molecular mechanisms. We first found that MYT1L loss increases the ratio of DL to UL neurons in the adult mouse cortex, consistent with the increase in expression of known DL genes observed in bulk RNA-seq. To understand how MYT1L might mediate this shift in neuronal proportion, and to understand the consequences of MYT1L loss on both gene expression and epigenome marks, we mapped high confidence MYT1L binding targets as well as histone landscapes using CUT&RUN on mouse brain samples. Integrating CUT&RUN data with existing ATAC-seq and RNA-seq data sets, we identified that MYT1L directly binds to a master regulator of DL fate, *Bcl11b* (Arlotta et al. 2005), and this gene was up-regulated following MYT1L loss in Het animals. With integrative investigations on MYT1L binding, chromatin accessibility, histone modifications, and gene expression, we also defined a novel role for MYT1L in the adult mouse brain in suppressing earlier neuronal development programs by closing chromatin structures and erasing active histone markers at binding sites, potentially via interactions with SIN3B and HDAC2. Our findings unravel MYT1L's role in repressing earlier neuronal development programs in the adult mouse brain and provide insights into long-term consequences of MYT1L loss during normal brain development.

**Figure 7.**
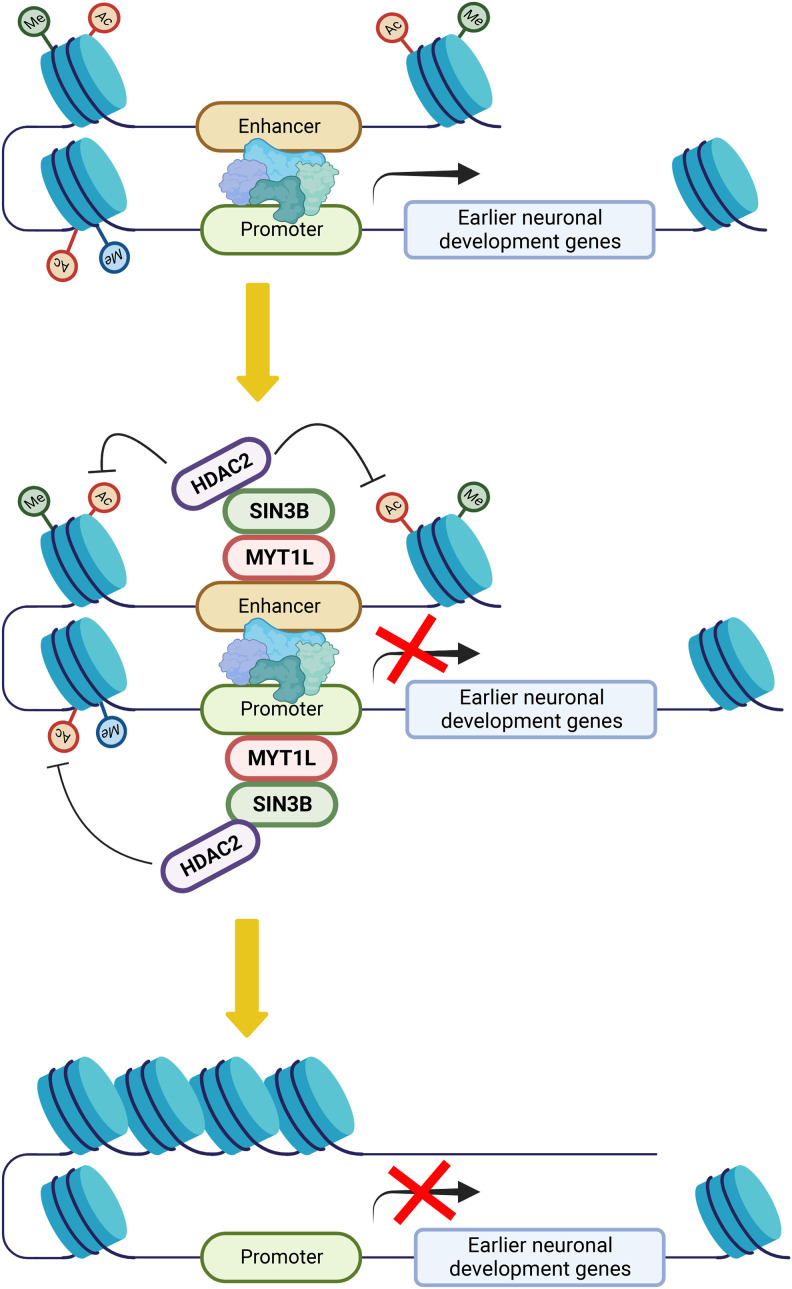
The model for MYT1L repressing earlier neuronal development programs to facilitate neuronal maturation.

MYT1L has been shown to repress non-neuronal genes to facilitate neuronal differentiation in MEF reprogramming. Integrating MYT1L CUT&RUN and multiomics data, we defined a novel role for MYT1L in repressing earlier neuronal development programs in the adult mouse cortex. We proposed that MYT1L normally binds to both promoter and enhancer regions in postmitotic neurons, recruits the SIN3B repressor complex containing HDAC2, and erases active histone marks to suppress earlier neuronal development genes (Fig. 7). Shutting down earlier neuronal programs may ensure postmitotic neurons maintain mature neuronal identity. Indeed, MYT1L Het PFC showed aberrant activation of promoters, enhancers, and thus gene expression associated with earlier neuronal development (Figs. 4–6; Supplemental Figs. S4–S7), suggesting neurons are trapped in an immature stage. This epigenetic alteration may explain the disrupted transcriptional, morphological, and electrophysiological properties previously observed (Chen et al. 2021).

In this study, CUT&RUN served as a powerful tool to profile MYT1L binding in vivo. Although promoters remain frequent targets of MYT1L, we found 56% of MYT1L targets are enhancers, which is a much higher percentage than in previously published ChIP-seq data (Mall et al. 2017). CUT&RUN is thought to have higher sensitivity and yield lower background signals compared with traditional ChIP-seq (Skene and Henikoff 2017). This may explain the larger number of MYT1L-bound enhancers detected here than prior ChIP-seq. In addition, the MYT1L KO samples provided key controls for optimizing the protocol and were not available at the time of the ChIP-seq experiments. MYT1L peaks identified by CUT&RUN were not present in KOs, suggesting CUT&RUN with this antibody was very specific for MYT1L (Fig. 2C). Consistent with this, the MYT1L core binding motif AAGTT has greater presence in peaks from CUT&RUN than ChIP-seq (76.4% vs. 32.1%). Meanwhile, CUT&RUN also exhibited different sensitivity between embryonic cortex and adult PFC, with many more MYT1L peaks called and more significant AAGTT motif enrichment from PFC (Supplemental Fig. S2D,E). Although most of the E14 peaks from both peak calling methods can be recovered in PFC experiments (Supplemental Fig. S2C,E), it is hard to tell if the unique peaks from two developmental time points are derived from differential bindings of MYT1L at the different ages, or rather a CUT&RUN sensitivity difference. CUT&RUN on more homogenous cell populations or single-cell TF-DNA interaction profiling assays (Cammack et al. 2020; Moudgil et al. 2020) will be needed to differentiate these two possibilities in the future. Likewise, how MYT1L influences histones and chromatin accessibility at its bound sites in E14 cortex requires further investigation.

MYT1L is a proneuronal TF that has been shown to regulate neuronal differentiation in many in vitro studies (Vierbuchen et al. 2010; Mall et al. 2017; Heavner et al. 2020). Here, utilizing *Myt1l* germline KO mice, we explored long-term consequences of MYT1L loss on neuronal molecular maturation in vivo. We found MYT1L levels influence neurons’ layer specific identity in the adult brain, whereas MYT1L mutation leads to increased DL neuron numbers and an up-regulation of the corresponding genes. This is consistent with the phenotype from a *Myt1l* shRNA knockdown study on primary cortical neuron cultures (Heavner et al. 2020). Another *Myt1l* shRNA knockdown experiment by in utero electroporation also showed that neurons with MYT1L loss fail to migrate into upper cortical layers during mouse embryonic development (Mall et al. 2017), which might eventually result in increased DL neuron numbers in the adult brain. CUT&RUN experiments revealed that MYT1L directly binds to genomic regions associated with earlier neuronal development, including neuronal migration and projection development, as well as the DL gene *Bcl11b*. Combining RNA-seq and ATAC-seq data sets, we proposed a role for MYT1L in providing proper suppression of these earlier neuronal development programs (ENDPs). We believe this can explain how MYT1L loss can simultaneously lead to precocious neuronal differentiation in embryos (and thus later microcephaly), yet prolonged neuronal immaturity in adults. Specifically, in embryos, MYT1L could normally help prevent premature expression of ENDPs. Thus, in mutant embryos, there is a bias in the progenitors to move too quickly from proliferation into earlier neuronal differentiation (Chen et al. 2021). Yet, once the animals pass the age at which these ENDPs are needed at a high level, they are also unable to recruit sufficient SIN3B and downstream HDACs to deacetylate many of these promoters and enhancers, and thus turn down the ENDPs to allow the neurons to complete their maturation. It is also possible that in addition to any effects of BCL11B on DL number directly, increased DL neuronal gene expression pattern might represent another aspect of the precocious differentiation and immaturity because DL neurons belong to an earlier neurodevelopmental trajectory than UL neurons in the cortex, which forms in an inside-out pattern during development (Shepherd and Rowe 2017).

Despite consensus findings on MYT1L's role in facilitating neuronal differentiation both in vitro and now in vivo, how MYT1L does so remains poorly understood. Unlike the repressive effects of MYT1L overexpression on non-neuronal genes described in the transdifferentiation system (Mall et al. 2017), we saw no obvious activation of non-neuronal genes on MYT1L loss (Chen et al. 2021). Likewise, we observed no obvious MYT1L binding at promoters of non-neural genes (e.g., liver, fibroblasts) in the CUT&RUN in vivo, in contrast to the ChIP-seq data in transdifferentiating cells (Mall et al. 2017). In addition, when overlapping MYT1L CUT&RUN targets with ChIP-seq targets from MEFs overexpressing MYT1L, we saw minimal shared hits between the two. Identical effects on different targets between in vitro and in vivo systems indicate MYT1L's repressive functions are context dependent, and ectopic expression of MYT1L might change its functions from those under physiological conditions. Likewise, MYT1L adult PFC CUT&RUN targets do not perfectly overlap with E14 CTX CUT&RUN or ChIP-seq targets but with shared strong hits (Supplemental Figs. S1, S2), suggesting an age-dependent binding activity of MYT1L.

In addition to functioning as a transcriptional repressor, evidence has been accumulated to suggest MYT1L can also activate transcription. We found MYT1L loss decreases its bound promoters’ accessibility via ATAC-seq when defining targeting using the older ChIP-seq in vitro data (Mall et al. 2017; Chen et al. 2021), consistent with loss of an activator. However, when conducting the ATAC-seq integration with the CUT&RUN defined MYT1L peaks, the data were more consistent with loss of a repressor (Fig. 5D). Yet, whereas on average the DARs and DEGs at MYT1L-bound genes tended to indicate it acts more often a repressor in WT brain, many individual loci showed a response in mutants more consistent with a loss of an activator (Figs. 4F–G, 5F–H; Supplemental Figs. S6G, S7G). Indeed, the N-terminus of MYT1L alone is sufficient to activate transcription in the luciferase reporter assay in vitro (Manukyan et al. 2018). We found those down-regulated loci are closely associated with mature neuronal functions, like ion channels and synaptic transmission (Fig. 5H; Supplemental Fig. S6C), suggesting MYT1L can also directly activate mature neuronal function genes. To explain these two faces of MYT1L, a “ready-set-go” model has been proposed, in which MYT1L cooperates with different cofactors to control neuronal gene transcription (Chen et al. 2022). Our study identified several cofactor candidates for MYT1L in vivo, including both transcriptional activators (SP1 and ELK1) and repressors (SIN3B), providing important hints for developing future models of how MYT1L tunes neuronal gene expression at different gene classes. High-throughput techniques, including massive parallel reporter assays (MPRA) (Mulvey et al. 2021), can be leveraged in the future to further examine the motif and cofactor requirements at MYT1L targets for repression and activation, respectively.

We also noticed that not all targets bound by MYT1L responded with a uniform magnitude to *Myt1l* heterozygosity, which may shed some light on the phenotype in *Myt1l* Het mice and haploinsufficient patients. Determining why certain MYT1L-bound genes are specifically sensitive to MYT1L levels is another important future direction. We noted that neurite outgrowth genes, which are often disrupted on MYT1L loss (Figs. 5H, 6C), may be more dependent on neural activity-dependent gene expression than other processes. Given MYT1L is often cobinding with activity-dependent genes like FOS and JUN at enhancers, it may be that MYT1L is needed to turn off activity-dependent signals after their activation. If so, this would fit with an earlier theory suggesting intellectual disability (ID) and autism spectrum conditions (ASCs) may be a general consequence of mistimed activity-dependent gene expression (Ebert and Greenberg 2013), though this would require further manipulations to assess in MYT1L Syndrome models.

Collectively, we mapped MYT1L binding targets via CUT&RUN and defined a function in suppressing ENDPs in the adult mouse brain. In addition, the data here should provide a foundation to study how cobinding partners and/or neural activity might influence the function of MYT1L in gene expression and histone modification. Such detailed investigations on MYT1L functions both in the WT and the MYT1L Syndrome mouse model could advance our understanding of the complicated progression of neuronal development in both physiological and pathological conditions.

## Methods

### Animal models

All procedures using mice were approved by the Institutional Care and Use Committee at Washington University School of Medicine. All mice used in this study were bred and maintained in the vivarium at Washington University in St. Louis in individually ventilated (36.2 × 17.1 × 13 cm) or static (28.5 × 17.5 × 12 cm; postweaning behavior only) translucent plastic cages with corncob bedding and ad libitum access to standard laboratory diet and water. Animals were kept at 12/12 h light/dark cycle, and room temperature (20°C–22°C) and relative humidity (50%) were controlled automatically. For all experiments, adequate measures were taken to minimize any pain or discomfort. Breeding pairs for experimental cohorts comprised *Myt1l* Hets and WT C57BL/6J mice (JAX Stock No. 000664) to generate male and female *Myt1l* Het and WT littermates. For embryonic CUT&RUN, *Myt1l* Het × Het breeding pairs were used to generate *Myt1l* WT and homozygous mutant littermates. Animals were weaned at P21, and group-housed by sex and genotype, until tissue harvest at P60 of age. Biological replicates for all experiments were sex and genotype balanced.

### Gene set enrichment analysis (GSEA)

GSEA was performed as described before (Subramanian et al. 2005) using GSEA v4.2.3 (https://www.gsea-msigdb.org/gsea/index.jsp). Deep layer neuron and upper layer neuron gene lists were obtained from Heavner et al. (2020). RNA-seq data sets on *Myt1l* germline KO mice were obtained from Chen et al. (2021). All analysis was performed with “gene_set” as permutation type and 1000 permutations. Significant enrichment was determined by FDR < 0.05 cutoff.

### Histopathology

Mice (5 WTs and 5 Hets for POU3F2 staining, 7 WTs and 6 Hets for BCL11B staining, sex balanced, at the age of P60) were deeply anesthetized and transcardially perfused with 4% paraformaldehyde in PBS. Whole brains were weighed and serially sectioned in the coronal plane at 75 μm using a vibratome and immunolabeled for either BCL11B (a marker for cortical layers V/VI) or POU3F2 (a marker for cortical layers II-IV). For each antibody, a set consisting of every eighth section was isolated and slide mounted. After drying overnight, antigen retrieval was performed by immersing in citrate buffer (pH 6.0) and pressure cooking for 10 min. The slides were then quenched in 3% hydrogen peroxide in absolute methanol for 10 min, immersed for 1 h in a blocking solution (2% bovine serum albumin, 0.2% dry milk, 0.8% Triton X-100 in PBS), and incubated overnight with a 1:500 dilution of either BCL11B (Abcam ab18465) or POU3F2 (Santa Cruz sc-393324). The next morning, BCL11B or POU3F2 incubated sections were reacted with appropriate biotinylated secondary antibody for 1 h (Sigma-Aldrich B7139; 1:200 or Vector Labs BA-9200; 1:200, respectively). The sections were then reacted with an avidin-biotin conjugate (ABC kit) for 1 h and visualized using the chromogen VIP (Vectastatin Elite ABC kit and Vector VIP kits; Vector Labs).

### Stereology

After immunolabeling, BCL11B or POU3F2 positive neurons were stereologically quantified using Stereoinvestigator Software (v 2019.1.3, MBF Bioscience) running on a Dell Precision Tower 5810 computer connected to a QImaging 2000R camera and a Labophot-2 Nikon microscope with electronically driven motorized stage. A rater, blind to treatment, stereologically quantified the number of positively stained cells using the unbiased optical fractionator method. To restrict counting to cortical regions with six layers, cell counts were performed on sections where the corpus callosum was visible and only in the neocortex (this excludes the allocortex, piriform, entorhinal, and retrosplenial cortices). Because each antibody labels specific cortical layers, volumes were calculated for only layers V-VI for BCL11B and I-IV for POU3F2 (POU3F2 did label cells in layers V-VI but these were not counted). Finally, a density was calculated by dividing the total number of positive cells by total volume for each antibody. Because maternal care, litter size, and other factors can cause litter effects, data were normalized by dividing each value by the average of the WT animals within each litter.

### CUT&RUN on embryonic and adult prefrontal cortex

CUT&RUN was performed on the embryonic and adult prefrontal cortex as previously described (Skene and Henikoff 2017; Brodie-Kommit et al. 2021). Three biological replicates were included for each age and genotype. Briefly, E14 mouse embryonic cortex or P60 mouse prefrontal cortex were dissected out, and nuclei were isolated using Nuclei EZ Prep Buffer (Sigma-Aldrich 4432370) and counted on cell cytometer. 300k nuclei were bound to the Concanavalin A-coated beads for each CUT&RUN reaction. Then, each aliquot of bead/nuclei were incubated with a primary antibody, including Rb-MYT1L (0.5 µg, Millipore ABE2915), Rb-H3K4me1 (1 µg, Abcam ab8895), Rb-H3K4me3 (1 µg, Active Motif 39159), Rb-H3K27ac (1 µg, Active Motif 39133), and Rb IgG (1 µg, Jackson ImmunoResearch 011-000-003), at 4°C on the nutator overnight. Next, to bind pAG-MNase fusion protein to the antibodies, beads were incubated with diluted CUTANA pAG-MNase (1:20, EpiCypher 15-1016) on the rotator at 4°C for 1 h. Chromatin digestion was performed at 0°C with the addition of CaCl_2_ (100 mM) for 30 min. To digest the RNA and release the cleaved DNA fragments, reactions were incubated with Stop Buffer at 37°C for 30 min in the thermocycler. Magnetic stands were used to bind beads afterwards, and supernants containing DNA fragments were retrieved for sequencing library preparation.

### CUT&RUN library preparation and Illumina sequencing

DNA fragments were extracted from CUT&RUN experimental supernatants by Phenol/Chloroform/Isoamyl Alcohol (pH 7.9) mix (Skene and Henikoff 2017). KAPA HyperPrep Kit (Roche KK8504) was used to generate dual-indexed sequencing libraries. Generated libraries were then purified using Mag-Bind beads. Finally, a robust nucleosome peaks pattern was confirmed as a quality control using an Agilent TapeStation and HS D1000 tapes. Finally, libraries were submitted to GTAC@MGI at Washington University School of Medicine for Illumina sequencing using a NovaSeq instrument, with a targeted read depth of 50 M reads per MYT1L and IgG library and 10 M reads per histone library.

### CUT&RUN data analysis

Raw reads were trimmed by Trimmomatic software to remove adapter sequence. FastQC was used to check read quality before and after trimming. Then reads were mapped to the mm10 genome by Bowtie 2 (Langmead and Salzberg 2012). Mitochondrial reads (SAMtools), PCR duplicates (Picard, https://broadinstitute.github.io/picard/), non-unique alignments (MAPQ > 30), and unmapped reads (SAMtools) (Li et al. 2009) were filtered out. MYT1L peaks were called from both individual biological replicates (q < 0.05) as well as from merged BAM files (merged by genotype, q < 0.01) MACS2 using IgG as background. Histones’ peaks were called from merged BAM files by MACS2 (q < 0.05) (Zhang et al. 2008) using down-sampled IgG as background. With MYT1L peaks called from individual biological replicates (high stringency), BEDTools (Quinlan and Hall 2010) was used to find intersecting peaks among 3 replicates, which allowed us to identify 560 peaks from E14 and 28,798 peaks from adult PFC CUT&RUN (Supplemental Table S1). With MYT1L peaks called from merged BAM files (low stringency), BEDTools was also used to exclude the 208 peaks found in KO samples, and this method resulted in 20,305 peaks from E14 CTX and 115,159 peaks from PFC CUT&RUN (Supplemental Table S1). Quality control analysis was performed to compare two peak calling methods, including peak enrichment distribution and HOMER de novo motif finding (Heinz et al. 2010). Due to better peak enrichment and MYT1L motif enrichment, only peaks from intersecting biological replicates were used for the downstream analysis, and both peak sets were provided in Supplemental Table 1. Next, these peaks were annotated by HOMER and then grouped into subcategories as described in the next section. Peak heatmaps were generated by the BEDTools plotHeatmap function. Genome track graphs were generated using Integrative Genomics Viewer (IGV; https://igv.org/) (Robinson et al. 2011). In order to compare changes of histone levels between WT and Het PFC, read counts within MYT1L CUT&RUN peaks were derived from individual histone CUT&RUN BAM files using BEDTools. Then, read counts were normalized to corresponding library sequencing depth and the average coverage was calculated from biological replicates within the genotype.

### Differential enrichment analysis for histone modifications

For each histone peak called, read counts were obtained by deepTools v3.5.0 (Ramírez et al. 2016) from individual biological replicates. Read counts were normalized by R package RUVSeq (r = 1) to remove unwanted variables and batch effects (Risso et al. 2014). Then, edgeR (Robinson et al. 2010) was used to perform differential enrichment analysis for histone peak read counts between WT and Het samples. Peaks showing FDR < 0.1 were defined as differentially enriched regions for the specific histone modification. Full results can be found in Supplemental Table S6.

### Definition of active and poised enhancers

Active and poised enhancers were defined as previously described (Creyghton et al. 2010). Briefly, enhancers were defined as H3K4me1 peaks located outside of the promoter regions (TSS ± 1 kb), with an absence of H3K4me3. Enhancers that overlap with H3K27ac peaks were categorized as active enhancers, and those without H3K27ac were defined as poised enhancers.

### Motif analysis

De novo motif discovery was performed using both HOMER and monaLisa (Machlab et al. 2022). For HOMER usage, full-length peaks were fed into the software, and ATAC-seq peaks from the same brain region and the same age were used as background. For monaLisa usage, promoter, active enhancer, and poised enhancer targets were grouped into separate bins and tested individually. To avoid length bias, peaks were resized into fixed-size regions around the peak midpoint before running the analysis. Then, the *k*-mer enrichment analysis was performed using monaLisa to examine the MYT1L core binding motif enrichment using 5 as unbiased motif length and ATAC-seq peaks as background. Finally, known motif finding was performed using monaLisa and the JASPAR2020 motif database on MYT1L-bound peaks. plotMotifHeatmaps was used to visualize significantly enriched known motifs with FDR < 1 × 10^−5^, and with TFs showing expression in PFC RNA-seq data set were plotted in the heatmap (Fig. 4A). To validate the motif analysis, different TFs’ ChIP-seq data from most relevant tissue types were acquired from the ChIP-Atlas (https://chip-atlas.org/) (Oki et al. 2018). Specifically, SP1 ChIP-seq on striatal neurons, ELK1 ChIP-seq on striatal neurons, JUNB ChIP-seq on activated cortical neurons, MEF2A ChIP-seq on PFC, NEUROD1 ChIP-seq on striatal neurons, and NEUROD2 ChIP-seq on striatal neurons cortex were fed into ChIPpeakAnno Package (Zhu et al. 2010) to overlap with MYT1L CUT&RUN data with a maximum gap of 200 bp.

### Predicting enhancer–gene pairs

Enhancer–gene pairs were predicted using EnhancerAtlas 2.0 (Gao and Qian 2020) (http://www.enhanceratlas.org/). Specifically, E14.5 Brain, Brain, and Neuron database were selected to annotate different subgroups of enhancers with their putative targeting genes.

### Gene Ontology (GO) analysis

GO analysis was performed using the BiNGO app in Cytoscape. *P*-values were adjusted by Benjamini–Hochberg FDR correction, and FDR < 0.05 cutoff was used to determine significant enrichments. Full GO analysis results can be seen in Supplemental Table S4.

### Integration of CUT&RUN and ATAC-seq data sets

ATAC-seq data sets, including peak files and differential accessible region analysis results, were obtained from Chen et al. (2021). The ChIPpeakAnno package was used to find overlapping peaks among MYT1L CUT&RUN, histones’ CUT&RUN, and ATAC-seq data sets. The maxgap value was set as 0. Then, fold change values for histone enrichments and ATAC-seq signals were retrieved for MYT1L-bound and -unbound regions, respectively. Boxplots were generated using R built-in functions.

### Coimmunoprecipitation

WT C57BL/6J P1 mouse cortex was dissected out in cold PBS and put into the lysis buffer (150 mM NaCl, 50 mM Tris, 1% Triton X-100) with protease inhibitors for homogenization. Brain lysates were centrifuged at 15,000×*g* for 10 min at 4°C. Then, supernatants were precleared with Protein A or G Dynabeads (Invirtogen 10006D) for 1 h at 4°C. Precleared lysates were used as immunoprecipitate inputs. To bind antibodies to beads, 1–5 μg Rb-MYT1L antibody was added into a 200 μL lysis buffer with 20 μL Dynabeads and rotated for 20 min at RT. Rabbit anti MYT1L (Proteintech 25234-1-AP) and Rabbit IgG (Jackson ImmunoResearch 011-000-003) were used for immunoprecipitation. The bead-antibody complex was washed twice with lysis buffer and then incubated with 100 μL of precleared brain lysates rotated at 4°C overnight. The bead-antibody-antigen complex then was washed four times with lysis buffer and resuspended in 15 μL lysis buffer plus 15 μL 2× sample buffer. The mixture was boiled for 10 min, and supernatants were separated from beads using a magnetic stand. Supernatants were then subjected to immunoblotting experiments for detecting candidate cofactors as described (Chen et al. 2021). Ms-SIN3B (Santa Cruz sc-13145), Ms-HDAC1 (Santa Cruz sc-81598), Ms-HDAC2 (Santa Cruz sc-9959), Ms-HDAC3 (Santa Cruz sc-376957), Ms-HDAC4 (Santa Cruz sc-46672), Ms-MEF2A (Santa Cruz sc-17785), and Rb-MYT1L (Proteintech 25234-1-AP) for primary antibody blotting. Full blot images were provided in Supplemental Figure S8.

### Statistical analysis

Statistical analyses and data graphing were performed using GraphPad Prism (v.8.2.1), and R (v.4.0.0) (R Core Team 2023). Prior to analyses, data was screened for missing values and for the fit of distributions with assumptions underlying univariate analysis. Means and standard errors were computed for each measure. Analysis of variance (ANOVA), including repeated measures or mixed models, was used to analyze data where appropriate. One-sample *t*-tests were used to determine differences from chance. For data that did not fit univariate assumptions, nonparametric tests were used or transformations were applied. Fisher's exact tests were used to assess MYT1L-bound and -unbound DAR distributions. Mann–Whitney *U* test was used to examine gene expression, ATAC-seq signal, and histone enrichment differences among groups. Multiple pairwise comparisons were subjected to Bonferroni correction or Dunnett correction. Figure schematics were generated using BioRender. All statistical data can be found in Supplemental Table S7.

## Data access

The CUT&RUN raw reads as well as counts data from this study have been submitted to the NCBI Gene Expression Omnibus (GEO; https://www.ncbi.nlm.nih.gov/geo/) under accession number GSE222072. The code for analyzing Illumina sequencing, ATAC-seq, and RNA-seq data generated in this study is available as Supplemental Code and at Bitbucket (https://bitbucket.org/jdlabteam/myt1l-cut-run-paper/src/master/).

## Supplementary Material

Supplemental Material

## References

[GR277413CHEC1] Almazan G, Lefebvre DL, Zingg HH. 1989. Ontogeny of hypothalamic vasopressin, oxytocin and somatostatin gene expression. Brain Res Dev Brain Res 45: 69–75. 10.1016/0165-3806(89)90008-4 2563676

[GR277413CHEC2] Arlotta P, Molyneaux BJ, Chen J, Inoue J, Kominami R, Macklis JD. 2005. Neuronal subtype-specific genes that control corticospinal motor neuron development in vivo. Neuron 45: 207–221. 10.1016/j.neuron.2004.12.036 15664173

[GR277413CHEC3] Arlotta P, Molyneaux BJ, Jabaudon D, Yoshida Y, Macklis JD. 2008. *Ctip2* controls the differentiation of medium spiny neurons and the establishment of the cellular architecture of the striatum. J Neurosci 28: 622–632. 10.1523/JNEUROSCI.2986-07.2008 18199763 PMC6670353

[GR277413CHEC4] Bainor AJ, Saini S, Calderon A, Casado-Polanco R, Giner-Ramirez B, Moncada C, Cantor DJ, Ernlund A, Litovchick L, David G. 2018. The HDAC-associated Sin3B protein represses DREAM complex targets and cooperates with APC/C to promote quiescence. Cell Rep 25: 2797–2807.e8. 10.1016/j.celrep.2018.11.024 30517867 PMC6324198

[GR277413CHEC5] Bedogni F, Hodge RD, Elsen GE, Nelson BR, Daza RAM, Beyer RP, Bammler TK, Rubenstein JLR, Hevner RF. 2010. Tbr1 regulates regional and laminar identity of postmitotic neurons in developing neocortex. Proc Natl Acad Sci 107: 13129–13134. 10.1073/pnas.1002285107 20615956 PMC2919950

[GR277413CHEC6] Black JC, Van Rechem C, Whetstine JR. 2012. Histone lysine methylation dynamics: establishment, regulation, and biological impact. Mol Cell 48: 491–507. 10.1016/j.molcel.2012.11.006 23200123 PMC3861058

[GR277413CHEC7] Blanchet P, Bebin M, Bruet S, Cooper GM, Thompson ML, Duban-Bedu B, Gerard B, Piton A, Suckno S, Deshpande C, 2017. *MYT1L* mutations cause intellectual disability and variable obesity by dysregulating gene expression and development of the neuroendocrine hypothalamus. PLoS Genet 13: e1006957. 10.1371/journal.pgen.1006957 28859103 PMC5597252

[GR277413CHEC8] Brodie-Kommit J, Clark BS, Shi Q, Shiau F, Kim DW, Langel J, Sheely C, Ruzycki PA, Fries M, Javed A, 2021. Atoh7-independent specification of retinal ganglion cell identity. Sci Adv 7: eabe4983. 10.1126/sciadv.abe4983 33712461 PMC7954457

[GR277413CHEC9] Cammack AJ, Moudgil A, Chen J, Vasek MJ, Shabsovich M, McCullough K, Yen A, Lagunas T, Maloney SE, He J, 2020. A viral toolkit for recording transcription factor–DNA interactions in live mouse tissues. Proc Natl Acad Sci 117: 10003–10014. 10.1073/pnas.1918241117 32300008 PMC7211997

[GR277413CHEC10] Campbell K. 2005. Cortical neuron specification: it has its time and place. Neuron 46: 373–376. 10.1016/j.neuron.2005.04.014 15882634

[GR277413CHEC11] Carullo NVN, Day JJ. 2019. Genomic enhancers in brain health and disease. Genes (Basel) 10: 43. 10.3390/genes10010043 30646598 PMC6357130

[GR277413CHEC12] Chen J, Lambo ME, Ge X, Dearborn JT, Liu Y, McCullough KB, Swift RG, Tabachnick DR, Tian L, Noguchi K, 2021. A MYT1L syndrome mouse model recapitulates patient phenotypes and reveals altered brain development due to disrupted neuronal maturation. Neuron 109: 3775–3792.e14. 10.1016/j.neuron.2021.09.009 34614421 PMC8668036

[GR277413CHEC13] Chen J, Yen A, Florian CP, Dougherty JD. 2022. MYT1L in the making: emerging insights on functions of a neurodevelopmental disorder gene. Transl Psychiatry 12: 292. 10.1038/s41398-022-02058-x 35869058 PMC9307810

[GR277413CHEC14] Coursimault J, Guerrot A-M, Morrow MM, Schramm C, Zamora FM, Shanmugham A, Liu S, Zou F, Bilan F, Le Guyader G, 2022. *MYT1L*-associated neurodevelopmental disorder: description of 40 new cases and literature review of clinical and molecular aspects. Hum Genet 141: 65–80. 10.1007/s00439-021-02383-z 34748075

[GR277413CHEC15] Creyghton MP, Cheng AW, Welstead GG, Kooistra T, Carey BW, Steine EJ, Hanna J, Lodato MA, Frampton GM, Sharp PA, 2010. Histone H3K27ac separates active from poised enhancers and predicts developmental state. Proc Natl Acad Sci 107: 21931–21936. 10.1073/pnas.1016071107 21106759 PMC3003124

[GR277413CHEC16] Dixit R, Wilkinson G, Cancino GI, Shaker T, Adnani L, Li S, Dennis D, Kurrasch D, Chan JA, Olson EC, 2014. *Neurog1* and *Neurog2* control two waves of neuronal differentiation in the piriform cortex. J Neurosci 34: 539–553. 10.1523/JNEUROSCI.0614-13.2014 24403153 PMC6608148

[GR277413CHEC17] Ebert DH, Greenberg ME. 2013. Activity-dependent neuronal signalling and autism spectrum disorder. Nature 493: 327–337. 10.1038/nature11860 23325215 PMC3576027

[GR277413CHEC18] Gao T, Qian J. 2020. EnhancerAtlas 2.0: an updated resource with enhancer annotation in 586 tissue/cell types across nine species. Nucleic Acids Res 48: D58–D64. 10.1093/nar/gkaa197 31740966 PMC7145677

[GR277413CHEC19] Götz M, Huttner WB. 2005. The cell biology of neurogenesis. Nat Rev Mol Cell Biol 6: 777–788. 10.1038/nrm1739 16314867

[GR277413CHEC20] Hayakawa T, Ohtani Y, Hayakawa N, Shinmyozu K, Saito M, Ishikawa F, Nakayama J. 2007. RBP2 is an MRG15 complex component and down-regulates intragenic histone H3 lysine 4 methylation. Genes Cells 12: 811–826. 10.1111/j.1365-2443.2007.01089.x 17573780

[GR277413CHEC21] Heavner WE, Ji S, Notwell JH, Dyer ES, Tseng AM, Birgmeier J, Yoo B, Bejerano G, McConnell SK. 2020. Transcription factor expression defines subclasses of developing projection neurons highly similar to single-cell RNA-seq subtypes. Proc Natl Acad Sci 117: 25074–25084. 10.1073/pnas.2008013117 32948690 PMC7547209

[GR277413CHEC22] Heintzman ND, Hon GC, Hawkins RD, Kheradpour P, Stark A, Harp LF, Ye Z, Lee LK, Stuart RK, Ching CW, 2009. Histone modifications at human enhancers reflect global cell-type-specific gene expression. Nature 459: 108–112. 10.1038/nature07829 19295514 PMC2910248

[GR277413CHEC23] Heinz S, Benner C, Spann N, Bertolino E, Lin YC, Laslo P, Cheng JX, Murre C, Singh H, Glass CK. 2010. Simple combinations of lineage-determining transcription factors prime *cis*-regulatory elements required for macrophage and B cell identities. Mol Cell 38: 576–589. 10.1016/j.molcel.2010.05.004 20513432 PMC2898526

[GR277413CHEC24] Heng JI-T, Chariot A, Nguyen L. 2010. Molecular layers underlying cytoskeletal remodelling during cortical development. Trends Neurosci 33: 38–47. 10.1016/j.tins.2009.09.003 19837469

[GR277413CHEC25] Jiang Y, Yu VC, Buchholz F, O'Connell S, Rhodes SJ, Candeloro C, Xia Y-R, Lusis AJ, Rosenfeld MG. 1996. A novel family of Cys-Cys, His-Cys zinc finger transcription factors expressed in developing nervous system and pituitary gland. J Biol Chem 271: 10723–10730. 10.1074/jbc.271.18.10723 8631881

[GR277413CHEC26] Kawauchi T, Hoshino M. 2008. Molecular pathways regulating cytoskeletal organization and morphological changes in migrating neurons. Dev Neurosci 30: 36–46. 10.1159/000109850 18075253

[GR277413CHEC27] Kim S, Oh H, Choi SH, Yoo Y-E, Noh YW, Cho Y, Im GH, Lee C, Oh Y, Yang E, 2022. Postnatal age-differential ASD-like transcriptomic, synaptic, and behavioral deficits in *Myt1l*-mutant mice. Cell Rep 40: 111398. 10.1016/j.celrep.2022.111398 36130507

[GR277413CHEC28] Kroon T, van Hugte E, van Linge L, Mansvelder HD, Meredith RM. 2019. Early postnatal development of pyramidal neurons across layers of the mouse medial prefrontal cortex. Sci Rep 9: 5037. 10.1038/s41598-019-41661-9 30911152 PMC6433913

[GR277413CHEC29] Langmead B, Salzberg SL. 2012. Fast gapped-read alignment with Bowtie 2. Nat Methods 9: 357–359. 10.1038/nmeth.1923 22388286 PMC3322381

[GR277413CHEC30] Li H, Handsaker B, Wysoker A, Fennell T, Ruan J, Homer N, Marth G, Abecasis G, Durbin R, 1000 Genome Project Data Processing Subgroup. 2009. The Sequence Alignment/Map format and SAMtools. Bioinformatics 25: 2078–2079. 10.1093/bioinformatics/btp352 19505943 PMC2723002

[GR277413CHEC31] Lomvardas S, Maniatis T. 2016. Histone and DNA modifications as regulators of neuronal development and function. Cold Spring Harb Perspect Biol 8: a024208. 10.1101/cshperspect.a024208 27371659 PMC4930923

[GR277413CHEC32] Lu L, Liu X, Huang W-K, Giusti-Rodríguez P, Cui J, Zhang S, Xu W, Wen Z, Ma S, Rosen JD, 2020. Robust Hi-C maps of enhancer-promoter interactions reveal the function of non-coding genome in neural development and diseases. Mol Cell 79: 521–534.e15. 10.1016/j.molcel.2020.06.007 32592681 PMC7415676

[GR277413CHEC33] Luo L, O'Leary DDM. 2005. Axon retraction and degeneration in development and disease. Annu Rev Neurosci 28: 127–156. 10.1146/annurev.neuro.28.061604.135632 16022592

[GR277413CHEC34] Machlab D, Burger L, Soneson C, Rijli FM, Schübeler D, Stadler MB. 2022. monaLisa: an R/Bioconductor package for identifying regulatory motifs. Bioinformatics 38: 2624–2625. 10.1093/bioinformatics/btac102 35199152 PMC9048699

[GR277413CHEC35] Malik AN, Vierbuchen T, Hemberg M, Rubin AA, Ling E, Couch CH, Stroud H, Spiegel I, Farh KK-H, Harmin DA, 2014. Genome-wide identification and characterization of functional neuronal activity–dependent enhancers. Nat Neurosci 17: 1330–1339. 10.1038/nn.3808 25195102 PMC4297619

[GR277413CHEC36] Mall M, Kareta MS, Chanda S, Ahlenius H, Perotti N, Zhou B, Grieder SD, Ge X, Drake S, Euong Ang C, 2017. Myt1l safeguards neuronal identity by actively repressing many non-neuronal fates. Nature 544: 245–249. 10.1038/nature21722 28379941 PMC11348803

[GR277413CHEC37] Manukyan A, Kowalczyk I, Melhuish TA, Lemiesz A, Wotton D. 2018. Analysis of transcriptional activity by the Myt1 and Myt1l transcription factors. J Cell Biochem 119: 4644–4655. 10.1002/jcb.26636 29291346

[GR277413CHEC38] Moudgil A, Wilkinson MN, Chen X, He J, Cammack AJ, Vasek MJ, Lagunas T, Qi Z, Lalli MA, Guo C, 2020. Self-reporting transposons enable simultaneous readout of gene expression and transcription factor binding in single cells. Cell 182: 992–1008.e21. 10.1016/j.cell.2020.06.037 32710817 PMC7510185

[GR277413CHEC39] Mulvey B, Lagunas T, Dougherty JD. 2021. Massively parallel reporter assays: defining functional psychiatric genetic variants across biological contexts. Biol Psychiatry 89: 76–89. 10.1016/j.biopsych.2020.06.011 32843144 PMC7938388

[GR277413CHEC40] Naruse Y, Aoki T, Kojima T, Mori N. 1999. Neural restrictive silencer factor recruits mSin3 and histone deacetylase complex to repress neuron-specific target genes. Proc Natl Acad Sci 96: 13691–13696. 10.1073/pnas.96.24.13691 10570134 PMC24126

[GR277413CHEC41] Nishibuchi G, Shibata Y, Hayakawa T, Hayakawa N, Ohtani Y, Sinmyozu K, Tagami H, Nakayama J. 2014. Physical and functional interactions between the histone H3K4 demethylase KDM5A and the nucleosome remodeling and deacetylase (NuRD) complex. J Biol Chem 289: 28956–28970. 10.1074/jbc.M114.573725 25190814 PMC4200253

[GR277413CHEC42] Nitarska J, Smith JG, Sherlock WT, Hillege MMG, Nott A, Barshop WD, Vashisht AA, Wohlschlegel JA, Mitter R, Riccio A. 2016. A functional switch of NuRD chromatin remodeling complex subunits regulates mouse cortical development. Cell Rep 17: 1683–1698. 10.1016/j.celrep.2016.10.022 27806305 PMC5149529

[GR277413CHEC43] Noack F, Vangelisti S, Raffl G, Carido M, Diwakar J, Chong F, Bonev B. 2022. Multimodal profiling of the transcriptional regulatory landscape of the developing mouse cortex identifies Neurog2 as a key epigenome remodeler. Nat Neurosci 25: 154–167. 10.1038/s41593-021-01002-4 35132236 PMC8825286

[GR277413CHEC44] Oki S, Ohta T, Shioi G, Hatanaka H, Ogasawara O, Okuda Y, Kawaji H, Nakaki R, Sese J, Meno C. 2018. ChIP-Atlas: a data-mining suite powered by full integration of public ChIP-seq data. EMBO Rep 19: e46255. 10.15252/embr.201846255 30413482 PMC6280645

[GR277413CHEC45] Olson JM, Asakura A, Snider L, Hawkes R, Strand A, Stoeck J, Hallahan A, Pritchard J, Tapscott SJ. 2001. NeuroD2 is necessary for development and survival of central nervous system neurons. Dev Biol 234: 174–187. 10.1006/dbio.2001.0245 11356028

[GR277413CHEC46] Pataskar A, Jung J, Smialowski P, Noack F, Calegari F, Straub T, Tiwari VK. 2016. NeuroD1 reprograms chromatin and transcription factor landscapes to induce the neuronal program. EMBO J 35: 24–45. 10.15252/embj.201591206 26516211 PMC4718003

[GR277413CHEC47] Quinlan AR, Hall IM. 2010. BEDTools: a flexible suite of utilities for comparing genomic features. Bioinformatics 26: 841–842. 10.1093/bioinformatics/btq033 20110278 PMC2832824

[GR277413CHEC048] Ramírez F, Ryan DP, Grüning B, Bhardwaj V, Kilpert F, Richter AS, Heyne S, Dündar F, Manke T. 2016. deepTools2: a next generation web server for deep-sequencing data analysis. Nucleic Acids Res 44: W160–W165. 10.1093/nar/gkw257 27079975 PMC4987876

[GR277413CHEC48] R Core Team. 2023. R: a language and environment for statistical computing. R Foundation for Statistical Computing, Vienna. https://www.R-project.org/.

[GR277413CHEC49] Risso D, Ngai J, Speed TP, Dudoit S. 2014. Normalization of RNA-seq data using factor analysis of control genes or samples. Nat Biotechnol 32: 896–902. 10.1038/nbt.2931 25150836 PMC4404308

[GR277413CHEC50] Robinson MD, McCarthy DJ, Smyth GK. 2010. edgeR: a Bioconductor package for differential expression analysis of digital gene expression data. Bioinformatics 26: 139–140. 10.1093/bioinformatics/btp616 19910308 PMC2796818

[GR277413CHEC51] Robinson JT, Thorvaldsdóttir H, Winckler W, Guttman M, Lander ES, Getz G, Mesirov JP. 2011. Integrative genomics viewer. Nat Biotechnol 29: 24–26. 10.1038/nbt.1754 21221095 PMC3346182

[GR277413CHEC52] Romm E, Nielsen JA, Kim JG, Hudson LD. 2005. Myt1 family recruits histone deacetylase to regulate neural transcription. J Neurochem 93: 1444–1453. 10.1111/j.1471-4159.2005.03131.x 15935060 PMC1201409

[GR277413CHEC53] Shepherd GM, Rowe TB. 2017. Neocortical lamination: insights from neuron types and evolutionary precursors. Front Neuroanat 11: 100. 10.3389/fnana.2017.00100 29163073 PMC5673976

[GR277413CHEC54] Skene PJ, Henikoff S. 2017. An efficient targeted nuclease strategy for high-resolution mapping of DNA binding sites. eLife 6: e21856. 10.7554/eLife.21856 28079019 PMC5310842

[GR277413CHEC55] Stiles J, Jernigan TL. 2010. The basics of brain development. Neuropsychol Rev 20: 327–348. 10.1007/s11065-010-9148-4 21042938 PMC2989000

[GR277413CHEC56] Subramanian A, Tamayo P, Mootha VK, Mukherjee S, Ebert BL, Gillette MA, Paulovich A, Pomeroy SL, Golub TR, Lander ES, 2005. Gene set enrichment analysis: a knowledge-based approach for interpreting genome-wide expression profiles. Proc Natl Acad Sci 102: 15545–15550. 10.1073/pnas.0506580102 16199517 PMC1239896

[GR277413CHEC57] Trevino AE, Sinnott-Armstrong N, Andersen J, Yoon S-J, Huber N, Pritchard JK, Chang HY, Greenleaf WJ, Pașca SP. 2020. Chromatin accessibility dynamics in a model of human forebrain development. Science 367: eaay1645. 10.1126/science.aay1645 31974223 PMC7313757

[GR277413CHEC58] Tutukova S, Tarabykin V, Hernandez-Miranda LR. 2021. The role of neurod genes in brain development, function, and disease. Front Mol Neurosci 14: 662774. 10.3389/fnmol.2021.662774 34177462 PMC8221396

[GR277413CHEC59] Vierbuchen T, Ostermeier A, Pang ZP, Kokubu Y, Südhof TC, Wernig M. 2010. Direct conversion of fibroblasts to functional neurons by defined factors. Nature 463: 1035–1041. 10.1038/nature08797 20107439 PMC2829121

[GR277413CHEC60] Wöhr M, Fong WM, Janas JA, Mall M, Thome C, Vangipuram M, Meng L, Südhof TC, Wernig M. 2022. *Myt1l* haploinsufficiency leads to obesity and multifaceted behavioral alterations in mice. Mol Autism 13: 19. 10.1186/s13229-022-00497-3 35538503 PMC9087967

[GR277413CHEC61] Yasumura A, Omori M, Fukuda A, Takahashi J, Yasumura Y, Nakagawa E, Koike T, Yamashita Y, Miyajima T, Koeda T, 2019. Age-related differences in frontal lobe function in children with ADHD. Brain Dev 41: 577–586. 10.1016/j.braindev.2019.03.006 30952459

[GR277413CHEC62] Yousefi S, Deng R, Lanko K, Salsench EM, Nikoncuk A, van der Linde HC, Perenthaler E, van Ham TJ, Mulugeta E, Barakat TS. 2021. Comprehensive multiomics integration identifies differentially active enhancers during human brain development with clinical relevance. Genome Med 13: 162. 10.1186/s13073-021-00980-1 34663447 PMC8524963

[GR277413CHEC63] Yuan W, Ma S, Brown JR, Kim K, Murek V, Trastulla L, Meissner A, Lodato S, Shetty AS, Levin JZ, 2022. Temporally divergent regulatory mechanisms govern neuronal diversification and maturation in the mouse and marmoset neocortex. Nat Neurosci 25: 1049–1058. 10.1038/s41593-022-01123-4 35915179 PMC9343253

[GR277413CHEC64] Zhang Y, Liu T, Meyer CA, Eeckhoute J, Johnson DS, Bernstein BE, Nusbaum C, Myers RM, Brown M, Li W, 2008. Model-based Analysis of ChIP-Seq (MACS). Genome Biol 9: R137. 10.1186/gb-2008-9-9-r137 18798982 PMC2592715

[GR277413CHEC65] Zhu LJ, Gazin C, Lawson ND, Pagès H, Lin SM, Lapointe DS, Green MR. 2010. ChIPpeakAnno: a Bioconductor package to annotate ChIP-seq and ChIP-chip data. BMC Bioinformatics 11: 237. 10.1186/1471-2105-11-237 20459804 PMC3098059

