# Supplementary material for: MYT1L is required for suppressing earlier neuronal development programs in the adult mouse brain
Source: Genome Res. 2023 Apr;33(4):541–56. doi: 10.1101/gr.277413.122 (PMC10234307; doi:10.1101/gr.277413.122)
Supplement: Supplemental Material [file supp_33_4_541__DC1.html]

MYT1L is required for suppressing earlier neuronal development programs in the adult mouse brain — MYT1L is required for suppressing earlier neuronal development programs in the adult mouse brain — Supplemental Material 

# MYT1L is required for suppressing earlier neuronal development programs in the adult mouse brain

## Supplemental Material

- Supplemental\_Table\_S2.xlsx
- Supplemental\_Table\_S3.xlsx
- Supplemental\_Table\_S4.xlsx
- Supplemental\_Table\_S5.xlsx
- Supplemental\_Table\_S6.xlsx
- Supplemental\_Table\_S7.xlsx
- Supplemental\_Table\_S1.xlsx
- Supplemental\_\_Figures.pdf
- Supplemental\_\_Code.zip
