# Supplementary material for: MYT1L is required for suppressing earlier neuronal development programs in the adult mouse brain
Source: Genome Res. 2023 Apr;33(4):541–56. doi: 10.1101/gr.277413.122 (PMC10234307; doi:10.1101/gr.277413.122)
Supplement: Supplemental Material [file supp_gr.277413.122_Supplemental__Figures.pdf]

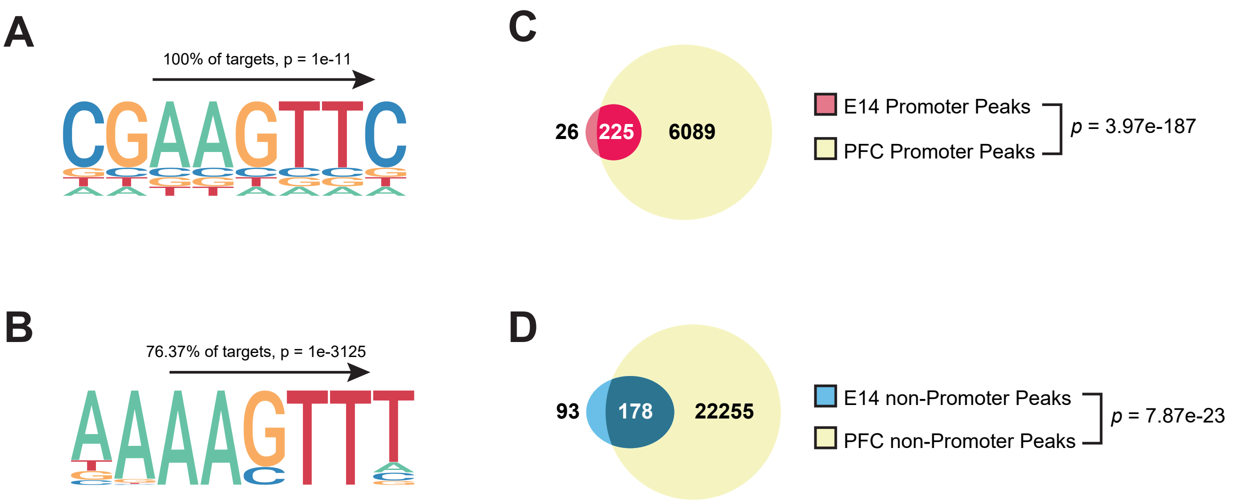

Supplemental Figure 1: CUT&RUN is more efficient for MYT1L binding profiling on P60 PFC than on E14 CTX. (**A**) Homer de novo motif finding shows significant enrichment of MYT1L core binding motif AAGTT in E14 MYT1L CUT&RUN peaks. (**B**) Homer de novo motif finding shows significant enrichment of MYT1L core binding motif AAGTT in PFC MYT1L CUT&RUN peaks. (**C**) Overlaps between MYT1L PFC and E14 CUT&RUN promoter peaks. (**D**) Overlaps between MYT1L PFC and E14 CUT&RUN non-promoter peaks.

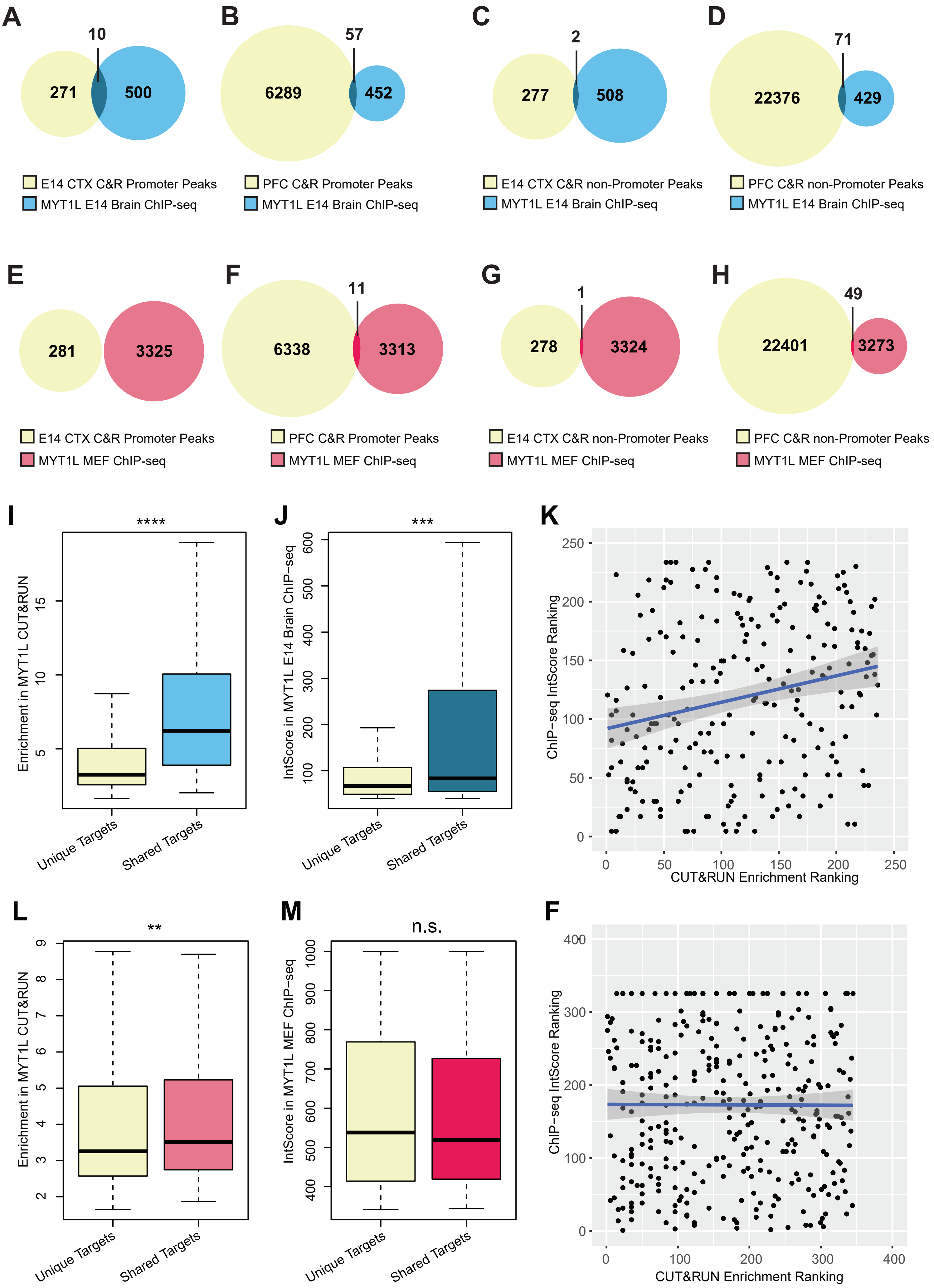

A
10
271
500
E14 CTX C&R Promoter Peaks
MYT1L E14 Brain ChIP-seq
B
57
6289
452
PFC C&R Promoter Peaks
MYT1L E14 Brain ChIP-seq
C
2
277
508
E14 CTX C&R non-Promoter Peaks
MYT1L E14 Brain ChIP-seq
D
71
22376
429
PFC C&R non-Promoter Peaks
MYT1L E14 Brain ChIP-seq
E
281
3325
E14 CTX C&R Promoter Peaks
MYT1L MEF ChIP-seq
F
11
6338
3313
PFC C&R Promoter Peaks
MYT1L MEF ChIP-seq
G
1
278
3324
E14 CTX C&R non-Promoter Peaks
MYT1L MEF ChIP-seq
H
49
22401
3273
PFC C&R non-Promoter Peaks
MYT1L MEF ChIP-seq
I
****
Enrichment in MYT1L CUT&RUN
Unique Targets
Shared Targets
J
***
IntScore in MYT1L E14 Brain ChIP-seq
Unique Targets
Shared Targets
K
ChIP-seq IntScore Ranking
CUT&RUN Enrichment Ranking
L
**
Enrichment in MYT1L CUT&RUN
Unique Targets
Shared Targets
M
n.s.
IntScore in MYT1L MEF ChIP-seq
Unique Targets
Shared Targets
F
ChIP-seq IntScore Ranking
CUT&RUN Enrichment Ranking

Supplemental Figure 2: MYT1L CUT&RUN on E14 CTX and adult PFC poorly overlaps with MYT1L ChIP-seq on E14 mouse brain and MEFs. (**A**) No significant overlap was found between MYT1L E14 CTX CUT&RUN promoter peaks and MYT1L E14 brain ChIP-seq peaks. (**B**) No significant overlap was found between MYT1L PFC CUT&RUN promoter peaks and MYT1L E14 brain ChIP-seq peaks. (**C**) No significant overlap was found between MYT1L E14 CTX CUT&RUN non-promoter peaks and MYT1L E14 brain ChIP-seq peaks. (**D**) No significant overlap was found between MYT1L PFC CUT&RUN non-promoter peaks and MYT1L E14 brain ChIP-seq peaks.(**E**) No significant overlap was found between MYT1L E14 CTX CUT&RUN promoter peaks and MYT1L MEF ChIP-seq peaks. (**F**) No significant overlap was found between MYT1L PFC CUT&RUN promoter peaks and MYT1L MEF ChIP-seq peaks. (**G**) No significant overlap was found between MYT1L E14 CTX CUT&RUN non-promoter peaks and MYT1L MEF ChIP-seq peaks. (**H**) No significant overlap was found between MYT1L PFC CUT&RUN non-promoter peaks and MYT1L MEF ChIP-seq peaks. (**I**) Boxplot of peak enrichment from MYT1L PFC CUT&RUN for shared targets between CUT&RUN and E14 brain ChIP-seq. (**J**) Boxplot of peak scores from MYT1L E14 brain ChIP-seq for shared targets between CUT&RUN and E14 brain ChIP-seq. (**K**) Spearman's rank correlation of shared targets in two datasets ($p = 0.00049$. rho = 0.22). (**L**) Boxplot of peak enrichment from MYT1L PFC CUT&RUN for shared targets between CUT&RUN and MEF ChIP-seq. (**M**) Boxplot of peak scores from MYT1L E14 brain ChIP-seq for shared targets between CUT&RUN and MEF ChIP-seq. (**N**) Spearman's rank correlation of shared targets in two datasets ($p = 0.94$. rho = -0.0037). *$p<0.05$, **$p<0.01$, ***$p<0.001$, ****$p<0.0001$.

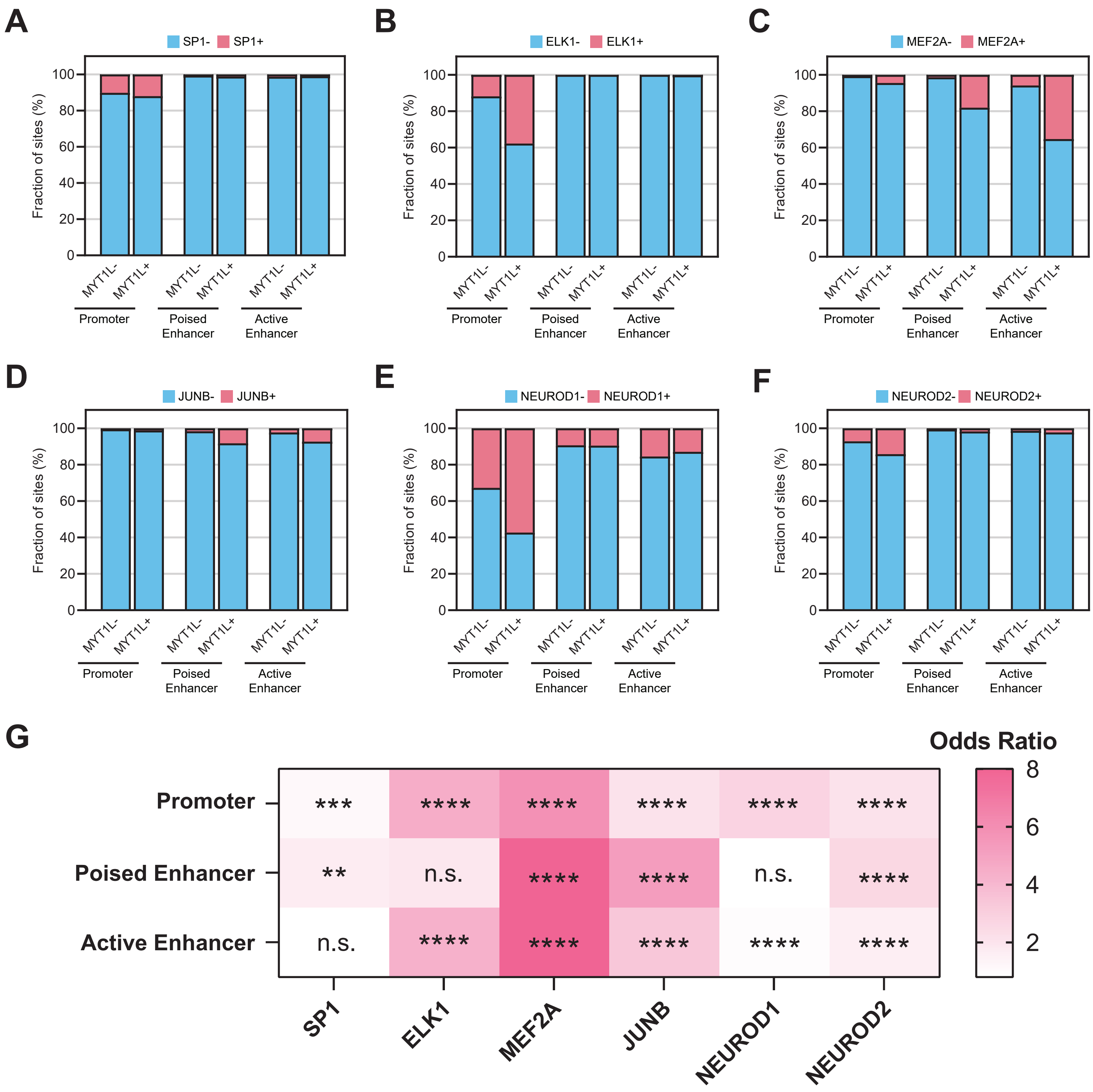

Supplemental Figure 3: MYT1L co-occupies with a different set of transcriptional factors at promoters and enhancers. (**A**) MYT1L+ promoters have a significantly higher percentage of SP1 binding compared to MYT1L- promoters, while no obvious difference was observed in enhancers. The same pattern was observed for (**B**) ELK1 as well. (**C**) MYT1L+ enhancers have a significantly higher percentage of MEF2A binding compared to MYT1L- promoters, while no big difference was observed in promoters. The same pattern was observed for (**D**) JUNB as well. (**E**) MYT1L+ promoters have a significantly higher percentage of NEUROD1 binding compared to MYT1L- promoters, while no obvious difference was observed in enhancers. The same pattern was observed for (**F**) NEUROD2 as well. (**G**) Fisher's exact test showed different TFs' enrichment in MYT1L+ over MYT1L- genomic regions. *p<0.05, **p<0.01, ***p<0.001, ****p<0.0001.

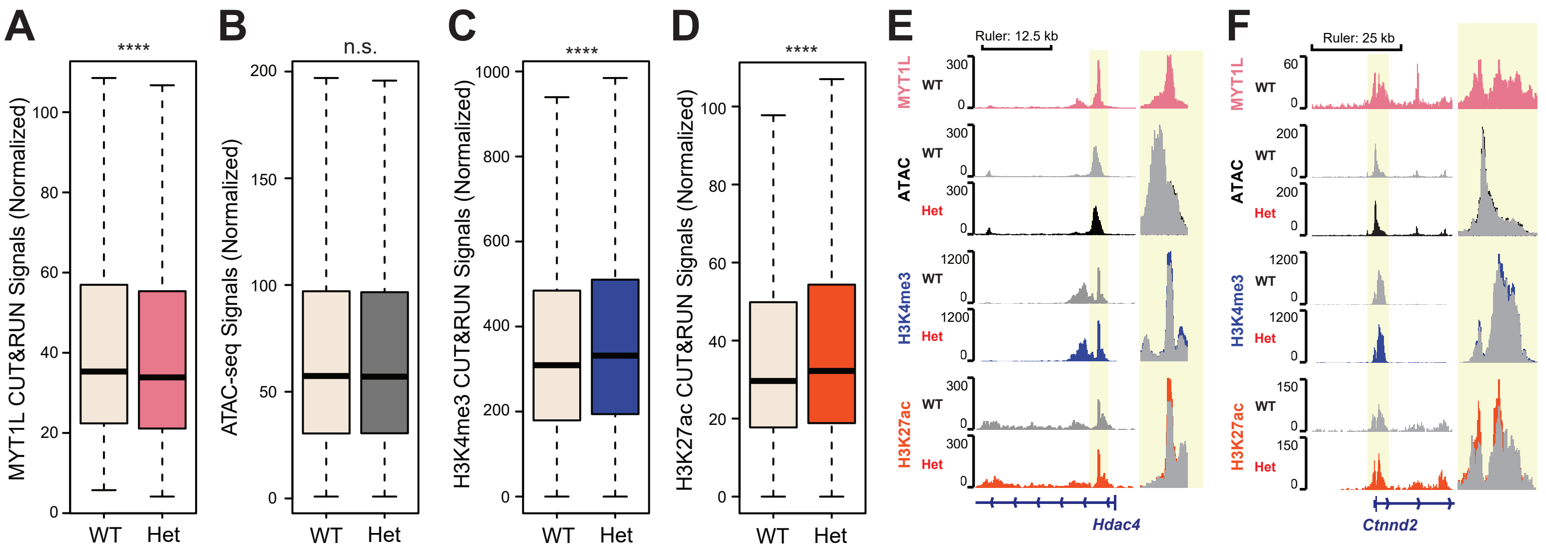

Supplemental Figure 4: MYT1L loss increases H3K4me3 and H3K27ac levels at promoters. (**A**) Het PFC showed decreased MYT1L CUT&RUN signals at MYT1L bound promoters. (**B**) MYT1L loss did not alter its bound promoters' chromatin accessibility. (**C**) MYT1L Het PFC has higher levels of H3K4me3 at MYT1L promoter targets compared to WTs. (**D**) MYT1L Het PFC has higher levels of H3K27ac at MYT1L promoter targets compared to WTs. (**E**) Representative genome browser track of MYT1L bound *Hdac4* promoter. (**F**) Representative genome browser track of MYT1L bound *Ctnnd2* promoter.
*$p<0.05$, **$p<0.01$, ***$p<0.001$, ****$p<0.0001$.

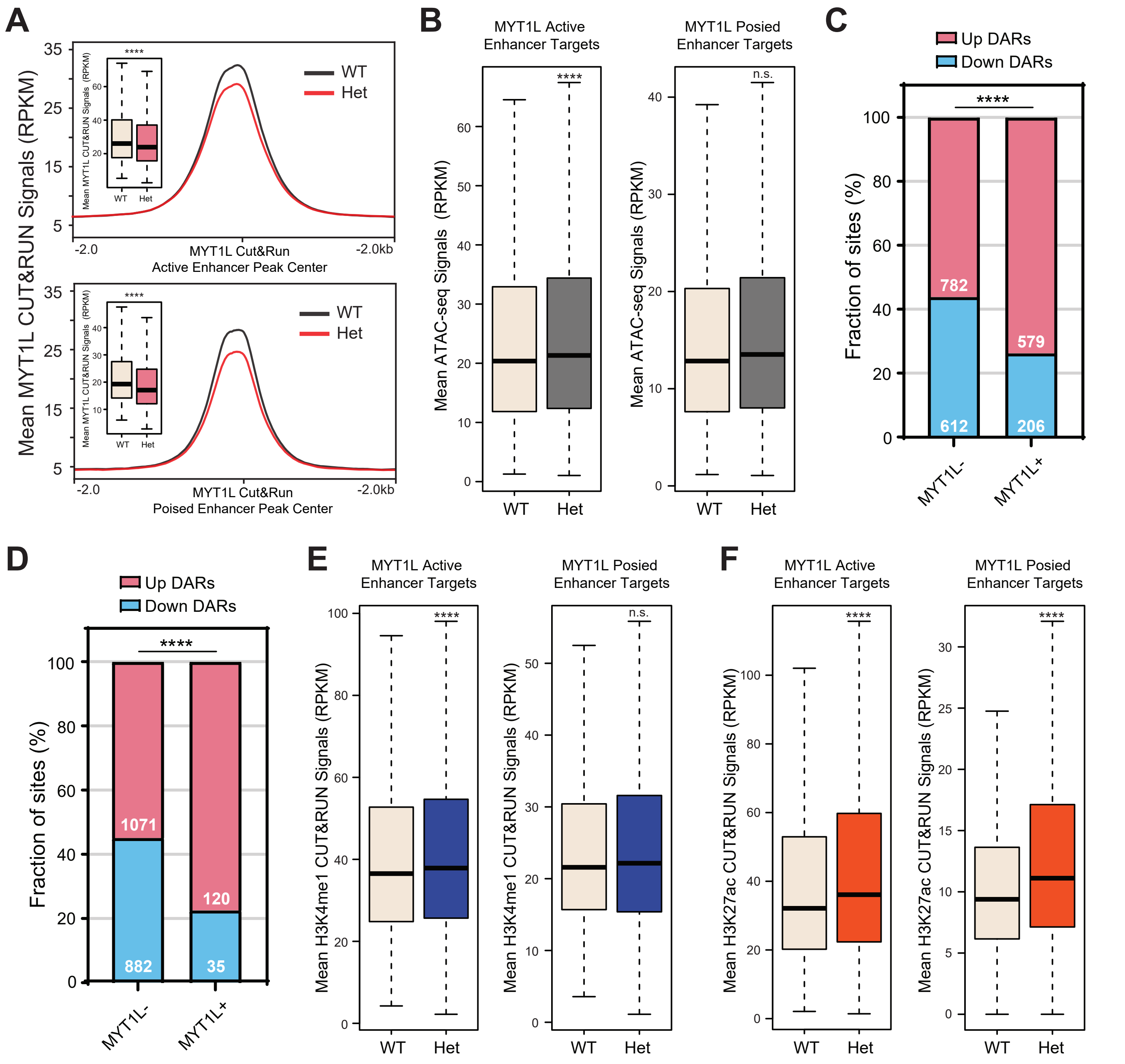

Supplemental Figure 5: MYT1L loss increases activation marks of its bound enhancers. (**A**) MYT1L Het PFC has reduced MYT1L binding at active (top) and poised (bottom) enhancers. (**B**) MYT1L loss increases its bound active (left) but not poised (right) enhancers' chromatin accessibility. (**C**) MYT1L bound active enhancer DARs have a higher percentage of DARs that increase accessibility than MYT1L unbound active enhancer DARs. (**D**) MYT1L bound poised enhancer DARs have a higher percentage of DARs that increase accessibility than MYT1L unbound poised enhancer DARs. (**E**) MYT1L Het PFC has higher levels of H3K4me1 at both active (left) and poised (right) enhancer targets compared to WTs. (**F**) MYT1L Het PFC has higher levels of H3K27ac at both active (left) and poised (right) enhancer targets compared to WTs. *p<0.05, **p<0.01, ***p<0.001, ****p<0.0001.

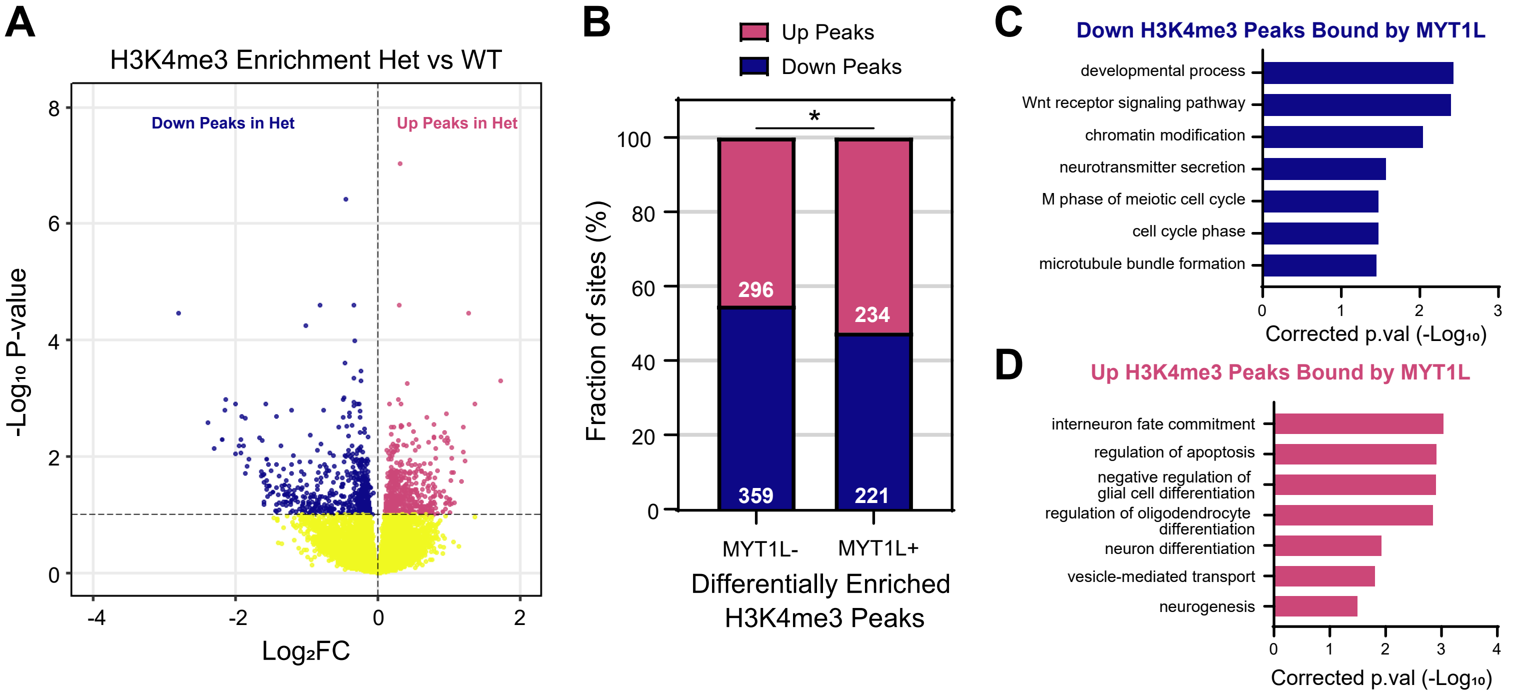

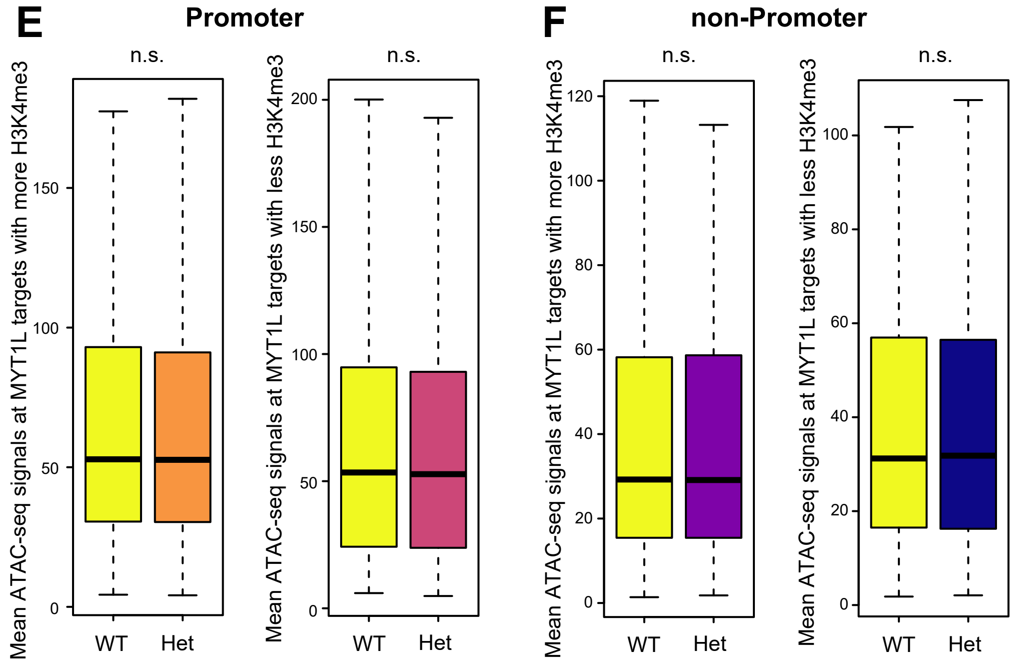

G

MYT1L Targets Showing Down-regulation in Both RNA-seq and H3K4me3	
**Gene**	**Functions**
Magee1	Regulation of transcription
Fbxo2	Promote cell proliferation
Fads1	Fatty acid metabolism

H

MYT1L Targets Showing Up-regulation in Both RNA-seq and H3K4me3	
**Gene**	**Functions**
Runx1t1	Transcriptional repressor, recruits deacetylase
Ago3	miRNA mediated gene silencing
Lingo1	Negative regulator of neuronal survival, axonal regeneration and oligodendrocyte differentiation
Per3	Circadian rhythm
Dlx2	Interneuron development
Lrrk2	GTP-GDP hydrolysis
Crebbp	Cell growth and division and differentiation

Supplemental Figure 6: MYT1L loss alters H3K4me3 landscape across the genome. (**A**) Volcano plot showing differential enrichment analysis identified 1110 diff-H3K4me3 peaks in Het PFC. (**B**) Distribution of down and up-regulated diff-H3K4me3 peaks within the MYT1L- and MYT1L+ categories. (**C**) GO analysis on down-regulated diff-H3K4me3 peaks bound by MYT1L and (**D**) up-regulated diff-H3K4me3 peaks bound by MYT1L. (**E**) Mean ATAC-seq signals of MYT1L+ diff-H3K4me3 promoter peaks (left: up-regulated peaks, right: down-regulated peaks) (**F**) Mean ATAC-seq signals of MYT1L+ diff-H3K4me3 non-promoter peaks (left: up-regulated peaks, right: down-regulated peaks). (**G**) Functions of MYT1L targets showing up-regulation in both RNA-seq and H3K4me3. *p<0.05, **p<0.01, ***p<0.001, ****p<0.0001.

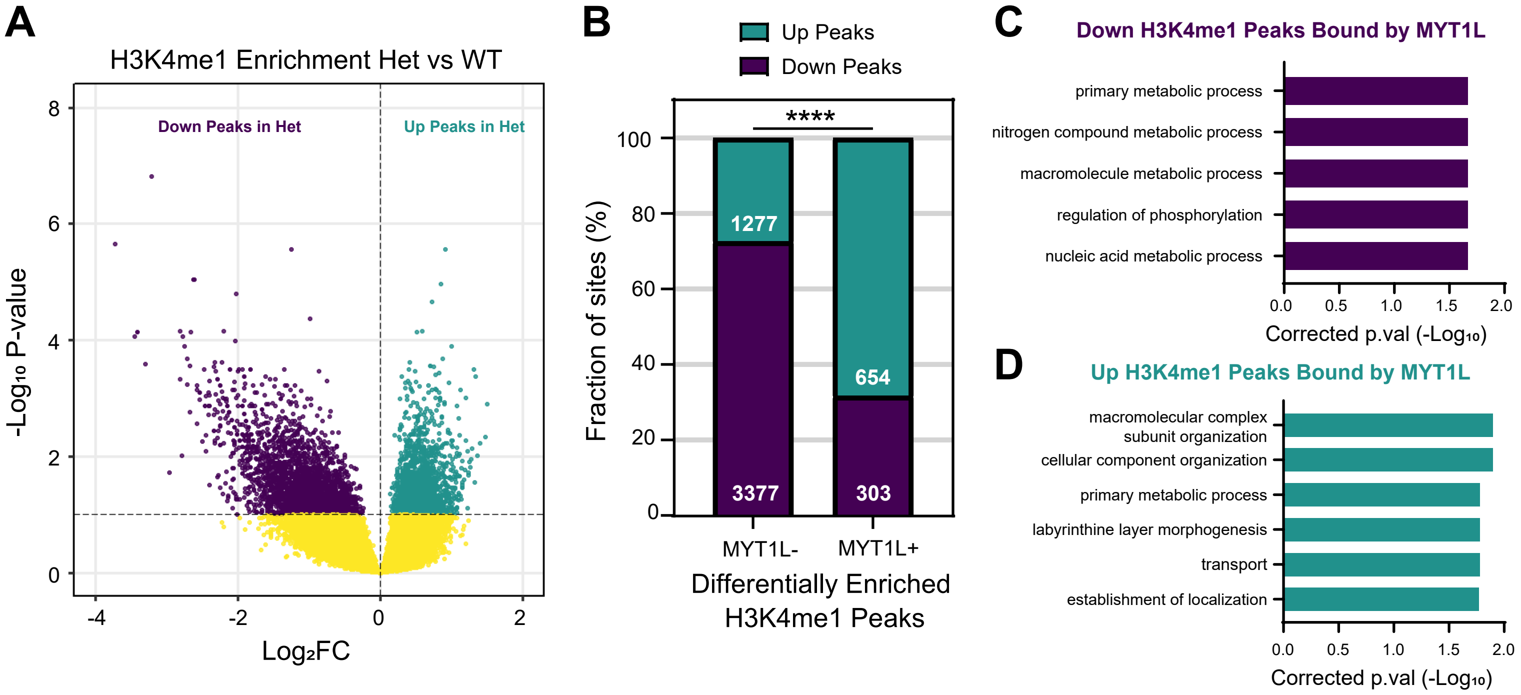

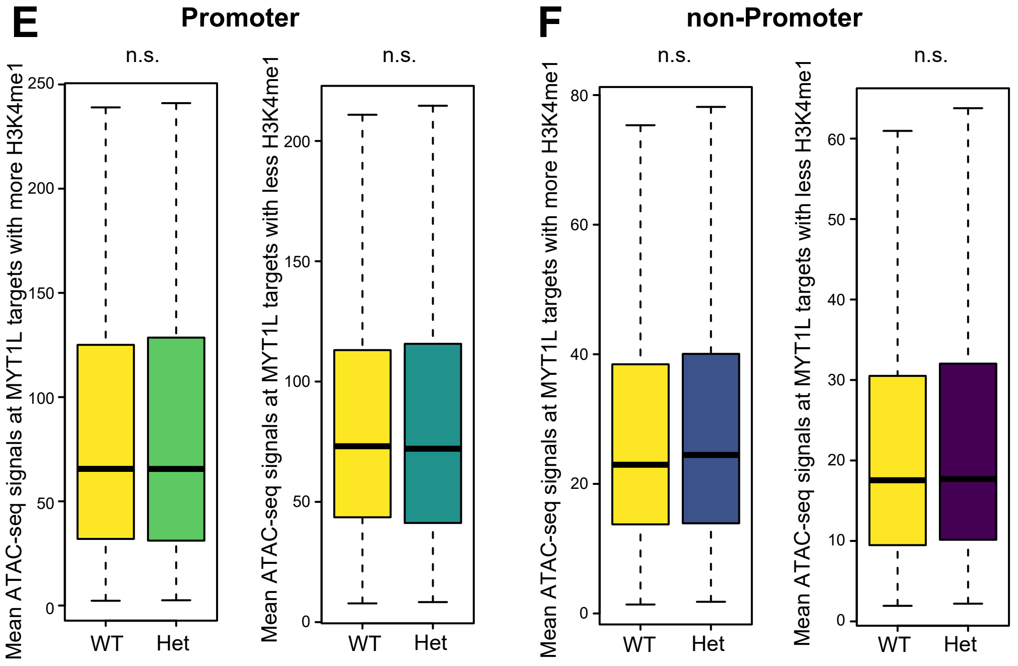

G

**MYT1L Targets Showing Down-regulation in Both RNA-seq and H3K4me1**	
**Gene**	**Functions**
Usp33	Promotes cell proliferation and reduces apoptosis

H

**MYT1L Targets Showing Up-regulation in Both RNA-seq and H3K4me1**	
**Gene**	**Functions**
Plekho1	Regulation of the actin cytoskeleton through its interactions with actin capping protein
Taok1	Controls cell viability and inflammation through activating downstream effectors such as NF-κB and mitogen-activated protein
Hmgn3	Reduce thes compactness of the chromatin fiber in nucleosomes, thereby enhancing transcription

Supplemental Figure 7: MYT1L loss alters H3K4me1 landscape across the genome. (**A**) Volcano plot showing differential enrichment analysis identified 1110 diff-H3K4me1 peaks in Het PFC. (**B**) Distribution of down and up-regulated diff-H3K4me1 peaks within the MYT1L- and MYT1L+ categories. (**C**) GO analysis on down-regulated diff-H3K4me1 peaks bound by MYT1L and (**D**) up-regulated diff-H3K4me1 peaks bound by MYT1L. (**E**) Mean ATAC-seq signals of MYT1L+ diff-H3K4me1 promoter peaks (left: up-regulated peaks, right: down-regulated peaks) (**F**) Mean ATAC-seq signals of MYT1L+ diff-H3K4me1 non-promoter peaks (left: up-regulated peaks, right: down-regulated peaks). (**G**) Functions of MYT1L targets showing up-regulation in both RNA-seq and H3K4me1. *p<0.05, **p<0.01, ***p<0.001, ****p<0.0001.

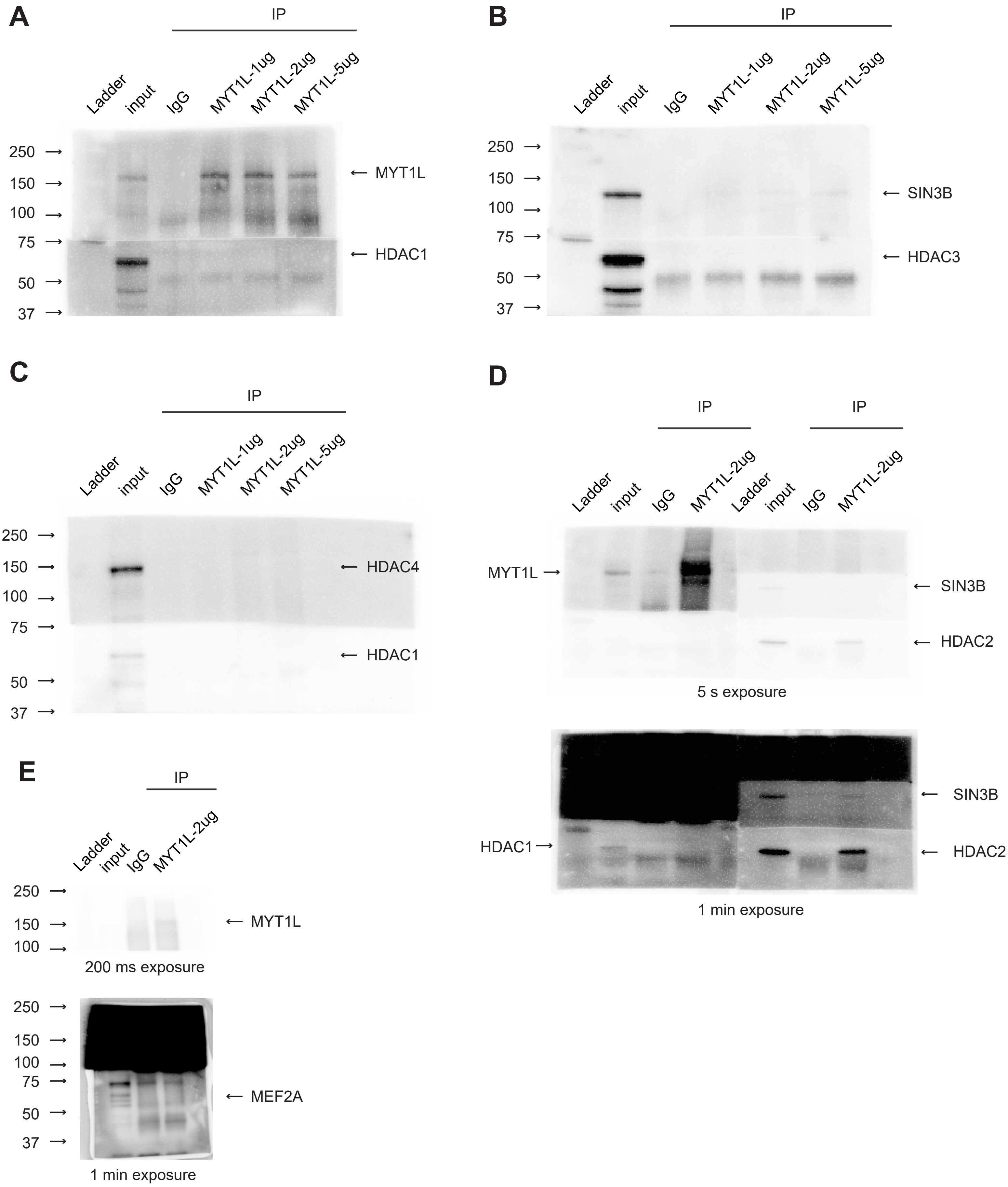

Supplemental Figure 8: Full western blot images of Co-IP experiments. (**A**) Co-IP shows MYT1L does not interact with HDAC1. (**B**) Co-IP shows MYT1L interacts with SIN3B but not HDAC4. (**C**) Co-IP shows MYT1L does not interact with HDAC1 and HDAC4. (**D**) Co-IP shows MYT1L interacts with SIN3B and HDAC2 but not HDAC1. (**E**) Co-IP shows MYT1L does not interact with MEF2A.
